# Inequalities in the provision of guideline-directed medical therapy following myocardial infarction: a cohort study

**DOI:** 10.1186/s12872-026-05572-8

**Published:** 2026-02-06

**Authors:** Fiona McLachlan, Marie de Bakker, Cesario Pancinha, Thomas M Caparrotta, Caroline Jackson, Thulani Ashcroft, Atul Anand, Peter J Gallacher, Eve Miller-Hodges, David Yeung, Neeraj Dhaun, Chris Tuck, Nicholas L Mills, Dorien M Kimenai

**Affiliations:** 1 Usher Institute, University of Edinburgh, Edinburgh, EH16 4UX UK; 2 British Heart Foundation Centre for Cardiovascular Science, University of Edinburgh, Edinburgh, UK; 3 Clinical Pharmacology Unit and Research Centre, University of Edinburgh, Edinburgh, UK; 4 Department of Renal Medicine, Royal Infirmary of Edinburgh, Edinburgh, UK

**Keywords:** Myocardial infarction, Secondary prevention, Treatment, Health inequalities

## Abstract

**Background:**

Following myocardial infarction (MI), therapies are recommended that reduce risk and prevent future cardiovascular events. Trends in the provision of guideline-directed medical therapies by sex, age, ethnicity and socioeconomic deprivation status may help identify opportunities to reduce inequalities in post-MI care.

**Methods:**

This cohort study using linked routine healthcare data included patients with MI in South-East Scotland (1 April 2009 to 31st of July 2021). Multivariable logistic regression models with a generalized estimating equation approach were used to assess the association between each sociodemographic factor and the provision of three guideline-directed medical therapies (anti-platelet or anti-thrombotic agent, lipid-lowering therapy and renin-angiotensin system blocker) at 3-, 12-, and 18-months post-discharge. Multivariable cause-specific Cox proportional hazard models were used to evaluate medication status and risk of a composite of non-fatal myocardial infarction, non-fatal stroke, or cardiovascular death across sociodemographic subgroups.

**Results:**

The study population comprised 7,926 patients (35% female, mean age 65 [SD 13] years). At 3 months, 5,393 (68%) patients were receiving all three guideline-directed medical therapies. Women (adjusted odds ratio at 3 months [aOR 0.69, 95% confidence interval 0.62 to 0.77]) and patients < 50 years (aOR 0.77, 95% confidence interval 0.65 to 0.89) and > 70 years (aOR 0.58, 95% confidence interval 0.51 to 0.65) were less likely to be receiving all three guideline-directed medical therapies at 3 months with similar observations at 12 and 18 months. No differences were observed by ethnicity and socioeconomic groups across the three time points. Patients receiving all three or one/two guideline-directed medical therapies had a lower risk of future cardiovascular events compared to those not receiving any, with this effect being consistent across all subgroups and time points.

**Conclusion:**

Women, and both younger and older patients are less likely to be receiving guideline-directed medical therapy following MI, despite its benefit in reducing future cardiovascular events. Targeted strategies to increase provision of secondary prevention in these groups are needed to reduce inequalities and improve post-MI care.

**Supplementary Information:**

The online version contains supplementary material available at 10.1186/s12872-026-05572-8.

## Background

Patients who survive acute myocardial infarction remain at risk of subsequent cardiovascular events [1, 2]. As such, clinical practice guidelines recommend preventative medication following myocardial infarction to reduce future cardiovascular risk with an anti-platelet or anti-thrombotic agent, lipid-lowering therapy and renin-angiotensin aldosterone (RAAS) blocker [3]. Variation in the use of secondary prevention following myocardial infarction by sex, age, ethnicity and socioeconomic deprivation status has been observed in recent decades [4–12].

Studies evaluating these disparities have often focused on the provision of statin therapy [5–8], and the initiation of guideline-directed medical therapies in hospital following myocardial infarction [4, 9–12]. A recent report from Thalmann et al*. *found that women, both younger and older patients and those with greatest socioeconomic deprivation were less likely to have a statin initiated within 150 days following myocardial infarction and were also more likely to discontinue treatment over time [5]. To date, it is unclear whether such inequalities apply in the use of other guideline-directed medical therapies and whether this observation persists over time. By identifying and understanding the determinants of the provision of guideline-directed medical therapies, prevention strategies can be tailored to better address the needs of all patients. Insights into the provision of guideline-directed medical therapies across these groups is important as it may help identifying opportunities to reduce inequalities in post-myocardial infarction care and clinical outcomes.

Our aim was to evaluate the provision of guideline-directed medical therapy over time in patients following myocardial infarction stratified by sex, age, ethnicity and socioeconomic deprivation, and to determine whether the use of guideline-directed medical therapy was associated with subsequent major adverse cardiovascular events (MACE) across these subgroups.

## Methods

### Study design and population

In this observational cohort study using routine healthcare data, we included all adult patients with a hospital record of acute myocardial infarction between the 1^st^ of April 2009 and the 31^st^ of July 2021, who were registered with a general practitioner practice in South-East Scotland, United Kingdom and survived to discharge. Incident episodes of myocardial infarction were identified from hospital discharge codes using the 10th revision of the International Classification of Diseases 10 (ICD-10) coding. The ICD-10 code I21 in the primary or secondary position was used to define myocardial infarction to optimize specificity [13]. Only patients with incident myocardial infarction were included, defined as patients with a hospital record of myocardial infarction and without a prior diagnosis of coronary heart disease up to 10-years before the date of the index diagnosis. Patients who died before discharge were excluded. All data were linked and de-identified by DataLoch (Edinburgh, United Kingdom) and analysed within their Trusted Research Environment. DataLoch (https://dataloch.org) is a data service developed in partnership by the University of Edinburgh and NHS Lothian Health Board, and provides a reliable source of electronic health records data, which has previously been used for evaluating patients with myocardial infarction [14–16]. The study was reviewed and received approval under delegated authority from the Institutional Review Board (REC 22/NS/0093) and Caldicott Guardian with a waiver for individual patient consent. This report followed the REporting of studies Conducted using Observational Routinely-collected Data (RECORD) guideline (Table S1).

### Data Sources

Data sources were linked through the Community Hospital Index (CHI), a unique identifier for patients living in Scotland. Information was extracted from the following data sources: the national Scottish Morbidity Record, National Records of Scotland, and Prescribing Information System, and the electronic patient record in primary care (Vision, Cegedim Healthcare Solutions, UK) and secondary care (TrakCare, InterSystems, Cambridge, MA). Data from the primary and secondary care electronic patient record were linked with biochemistry data, death records and community prescriptions. A five-year lookback period was employed to determine patient’s clinical characteristics including smoking status, body mass index, blood pressure, cholesterol concentrations, kidney function and electronic frailty index [17]. Comorbidities were determined by identifying relevant Read codes from primary care, as well as ICD-10 diagnostic codes from secondary care in the ten years preceding the incident myocardial infarction.

### Sociodemographic factors

The following sociodemographic factors were considered exposures: sex, age, ethnicity and socioeconomic deprivation status. Sex was categorised as women and men. Age was categorised as < 50 years, 50 to 70 years and > 70 years. Given the low ethnic diversity of our study population, ethnicity was categorised as White and Asian, Black, Mixed or other ethnicities. Socioeconomic deprivation status was determined using the Scottish Index of Multiple Deprivation 2016 (SIMD) score, which is a validated composite measure of socioeconomic deprivation based on a patient’s postcode [18], using the postcode closest to their index myocardial infarction date. Socioeconomic deprivation status was categorised as Group 1 (quintile 1 [representing the most deprived 20% of the population]), Group 2 (quintile 2 to 4) and Group 3 (quintile 5 [representing the least deprived 20% of the population]), as previously described [19].

### Guideline-directed medical therapy

Records of prescriptions for guideline-directed medical therapies, which were prescribed, processed, and submitted for payment, were identified through data linkage with the national Prescribing Information System. Our primary analysis included the following three drug classes: anti-platelet or anti-thrombotic agents, lipid-lowering therapy (statin and non-statins [ezetimibe or PCSK9 inhibitor]), and RAAS blockers (angiotensin converting enzyme inhibitors or angiotensin receptor blockers, Table S2). We evaluated the co-prescription of all three drug classes, the co-prescription of two or less than two drug classes or none. Given the evolving guideline recommendations for beta-blocker use following myocardial infarction in the United Kingdom, beta-blocker therapy is evaluated in a secondary analysis. Provision of guideline-directed therapy was evaluated at 3, 12 months and 18 months after discharge following the index event with a window of 45 days before and after these time periods.

### Cardiovascular outcomes

Cardiovascular outcomes were collected throughout the study period until the 1st of March 2023. The clinical outcome of interest was MACE including non-fatal myocardial infarction, non-fatal stroke, or cardiovascular death. These were defined using ICD-10 codes appearing in the first two positions of the hospitalization or death records: recurrent non-fatal myocardial infarction (ICD-10: I21-I22), non-fatal stroke (ICD-10 codes: I60, I61, I63, I64) or cardiovascular death (ICD-10: I00-I99).

### Statistical analysis

Continuous variables were presented as means and standard deviations (SDs) or median [25th percentile, 75th percentile], as appropriate. Categorical variables were presented as counts and percentages (%). Baseline characteristics were presented by the entire cohort, by treatment status at 3, 12 and 18 months after discharge and by subgroups.

The provision of all three guideline-directed medical therapies at 3-, 12-, and 18-months post-discharge was evaluated using logistic regression models with a generalized estimating equation approach. The dependent variable was guideline-directed medical therapy (all three guideline-directed medical therapies *versus* one/two or no guideline-medical directed therapy). Differences over time by subgroups were assessed through interaction terms between the subgroup of interest and time since discharge. Both univariable and multivariable logistic regression models were performed, with sociodemographic factors (sex, age, ethnicity and socioeconomic deprivation status) and electronic frailty index included as covariates in the multivariable models. Results are presented as the estimated probability (%) of receiving all three guideline-directed medical therapies in each subgroup as well as an odds ratio (95% confidence interval) compared to a reference group (e.g., women *versus* men).

The cumulative incidence of MACE at the median follow-up was estimated by guideline-directed medical therapy status and by subgroups. The association between guideline-directed medical therapy status and MACE was evaluated by subgroups groups after the 3-, 12-, and 18-month landmark point using cause-specific Cox proportional hazard regression models. The dependent variable was MACE. An interaction term for treatment status and the subgroup of interest was used to estimate associations in the subgroups separately. Both univariable and multivariable Cox regression models were performed, with sociodemographic factors (sex, age, ethnicity and socioeconomic deprivation status) and electronic frailty index included as covariates in the multivariable models.

A number of secondary analyses were conducted. We evaluated 1) the intersection of sex and age in the provision of guideline-directed medical therapies, 2) the provision of guideline-directed medical therapies in patients with and without coronary revascularization separately, 3) the impact of temporal changes on the provision of guideline-directed medical therapies by dividing the cohort in two periods (1st of April 2009—31st of March 2015 and 1st of April 2015–31st of July 2021), 4) the co-prescription of four drug classes: anti-platelet or anti-thrombotic agents, lipid-lowering therapy, RAAS blockers, and beta blockers and whether temporal differences in beta-blocker provision existed within our study population, and 5) the impact of comorbidities (i.e., renal function, and history of diabetes, heart failure, hypertension, obesity, stroke or transient ischemic attack) on the association between guideline-directed medical therapy status and MACE. Due to the exploratory nature of the analyses, we did not adjust for multiple testing. The proportion of missing values for each variable is reported in Table 1. Multiple imputation using the *mice* package was applied to account for missing values of covariates. Statistical analysis was performed in R version 4.4.2. The original R code for this study is available upon reasonable request.

**Table 1:** Baseline characteristics of patients with myocardial infarction by guideline-medical therapy status at 3 months

		**Guideline-directed medical therapy at 3 months**			
**Characteristic**	**Total** **n = 7926**	**Three** **n = 5393**	**One/two** **n = 2129**	**None** **n = 242**	**Died or censored** **n = 162**
Age (years)	64.5 (13.3)	62.5 (12.0)	68.3 (14.7)	69.2 (16.8)	73.9 (15.1)
Sex (% male)	5,124 (64.6%)	3,731 (69.2%)	1,166 (54.8%)	135 (55.8%)	92 (56.8%)
Ethnicity (%)					
White	7,033 (96.8%)	4,790 (96.8%)	1,908 (96.8%)	203 (95.8%)	132 (97.8%)
Asian/Black/Mixed/Other	235 (3.2%)	160 (3.2%)	63 (3.2%)	< 10	< 5
Socioeconomic deprivation* (%)					
Group 1 (most deprived)	1,354 (17.1%)	949 (17.6%)	338 (15.9%)	35 (14.5%)	32 (19.9%)
Group 2	4,620 (58.5%)	3,151 (58.6%)	1,226 (57.7%)	151 (62.7%)	92 (57.1%)
Group 3 (least deprived)	1,929 (24.4%)	1,277 (23.7%)	560 (26.4%)	55 (22.8%)	37 (23.0%)
History of diabetes (%)	1,238 (15.6%)	792 (14.7%)	370 (17.4%)	40 (16.5%)	36 (22.2%)
History of hypertension (%)	3,274 (41.3%)	2,141 (39.7%)	921 (43.3%)	120 (49.6%)	92 (56.8%)
History of obesity (%)	1,477 (18.6%)	1,009 (18.7%)	406 (19.1%)	34 (14.0%)	28 (17.3%)
History of stroke and/or TIA (%)	591 (7.5%)	296 (5.5%)	235 (11.0%)	38 (15.7%)	22 (13.6%)
History of heart failure (%)	311 (3.9%)	125 (2.3%)	143 (6.7%)	22 (9.1%)	21 (13.0%)
Current smoker (%)	2,539 (37.0%)	1,814 (39.3%)	608 (32.2%)	78 (37.5%)	39 (28.1%)
Body mass index (kg/m2)	28.0 [24.6, 31.7]	28.4 [25.2, 32.2]	27.0 [23.5, 30.8]	25.5 [22.3, 30.7]	25.5 [22.2, 30.5]
Systolic blood pressure (mmHg)	136.9 (18.1)	137.5 (17.7)	135.7 (18.4)	136.3 (22.6)	135.6 (17.6)
Diastolic blood pressure (mmHg)	79.5 (11.4)	80.3 (11.4)	77.7 (11.0)	77.8 (13.0)	76.6 (11.4)
Estimated glomerular filtration rate (% yes)					
< 30 ml/min/1.73m2	188 (2.4%)	44 (0.8%)	108 (5.2%)	16 (6.9%)	20 (12.7%)
30 to 44 ml/min/1.73m2	358 (4.6%)	138 (2.6%)	174 (8.3%)	30 (12.9%)	16 (10.2%)
45 to 59 ml/min/1.73m2	676 (8.6%)	388 (7.3%)	228 (10.9%)	28 (12.0%)	32 (20.4%)
≥ 60 ml/min/1.73m2	6,595 (84.4%)	4,764 (89.3%)	1,583 (75.6%)	159 (68.2%)	89 (56.7%)
Cholesterol (mmol/L)	5.1 (1.3)	5.2 (1.3)	5.1 (1.3)	4.7 (1.2)	4.6 (1.3)
Low-density lipoprotein cholesterol (mmol/L)	3.2 (1.1)	3.2 (1.1)	3.1 (1.1)	2.9 (1.1)	2.9 (1.2)
High-density lipoprotein cholesterol (mmol/L)	1.1 (0.3)	1.1 (0.3)	1.2 (0.4)	1.1 (0.4)	1.1 (0.4)
Triglycerides (mmol/L)	1.3 [1.0, 1.9]	1.4 [1.0, 1.9]	1.3 [0.9, 1.9]	1.3 [0.9, 1.8]	1.2 [0.9, 1.8]
Haemoglobin (g/L)	140.0 (18.5)	142.8 (16.9)	134.9 (19.9)	129.7 (22.4)	130.3 (21.8)
Glucose (mmol/L)	6.6 [5.6, 8.4]	6.7 [5.6, 8.3]	6.6 [5.5, 8.4]	6.6 [5.4, 8.6]	7.2 [5.9, 9.2]
Electronic frailty index** (%)					
No frailty	4,260 (62.5%)	3,159 (69.4%)	962 (50.1%)	93 (44.3%)	46 (34.1%)
Mild frailty	1,928 (28.3%)	1,128 (24.8%)	664 (34.6%)	77 (36.7%)	59 (43.7%)
Moderate frailty	518 (7.6%)	230 (5.0%)	234 (12.2%)	32 (15.2%)	22 (16.3%)
Severe frailty	115 (1.7%)	38 (0.8%)	61 (3.2%)	8 (3.8%)	8 (5.9%)
Medications prior to index event					
Antithrombotic agent (%)	1,349 (17.0%)	752 (13.9%)	499 (23.4%)	47 (19.4%)	51 (31.5%)
Lipid-lowering therapy (%)	1,813 (22.9%)	1,236 (22.9%)	487 (22.9%)	47 (19.4%)	43 (26.5%)
Renin-angiotensin system blocker (%)	1,895 (23.9%)	1,368 (25.4%)	428 (20.1%)	54 (22.3%)	45 (27.8%)
Index event characteristics					
Discharge diagnosis (%)					
STEMI	2,377 (39.5%)	1,905 (46.3%)	383 (23.8%)	45 (25.4%)	44 (36.4%)
NSTEMI	3,643 (60.5%)	2,211 (53.7%)	1,223 (76.2%)	132 (74.6%)	77 (63.6%)
Coronary artery bypass graft (%)	227 (2.9%)	110 (2.0%)	110 (5.2%)	< 5	< 5
Percutaneous coronary intervention (%)	5,150 (65.0%)	4,082 (75.7%)	943 (44.3%)	71 (29.3%)	54 (33.3%)

Continuous variables are presented as mean (standard deviation) or median [25th percentile, 75th percentile], as appropriate. Categorical variables are presented as number (%). Missing values < 5% if applicable, except for ethnicity (8.3%), current smoker (13.5%), body mass index (28.8%), systolic blood pressure (10.8%), diastolic blood pressure (10.8%), cholesterol (23.2%), low-density lipoprotein (36.2%), high-density lipoprotein (34.6%), triglycerides (33.9%), glucose (18.9%), electronic frailty index (13.9%), and discharge diagnosis (24.0%)

*STEMI* ST segment elevation myocardial infarction, *NSTEMI* non-ST segment elevation myocardial infarction, *TIA* transient ischaemic attack

^*^Socioeconomic deprivation is determined using the Scottish Index of Multiple Deprivation (SIMD). The SIMD is an area-based measure of relative deprivation. Patients were assigned a quintile based on their individual SIMD rank and were divided into three subgroups: Group 1 (quintile 1) representing the most deprived 20% of the population, Group 2 (quintile 2 to 4), and Group 3 (quintile 5) representing the least deprived 20% of the population

^**^The electronic frailty index is a tool that uses routinely collected primary care data to assess and quantify an individual's level of frailty based on the accumulation of health deficits. A higher score on the electronic frailty index indicates a higher level of frailty. Patients were divided into 4 groups based on their electronic frailty index: Fit or no frailty (frailty index 0 to 0.12), Mild frailty (frailty index > 0.12 to 0.24), Moderate frailty (frailty index > 0.24 to 0.36), and Severe frailty (frailty index > 0.36)

## Results

### Study population

**Fig. 1:**
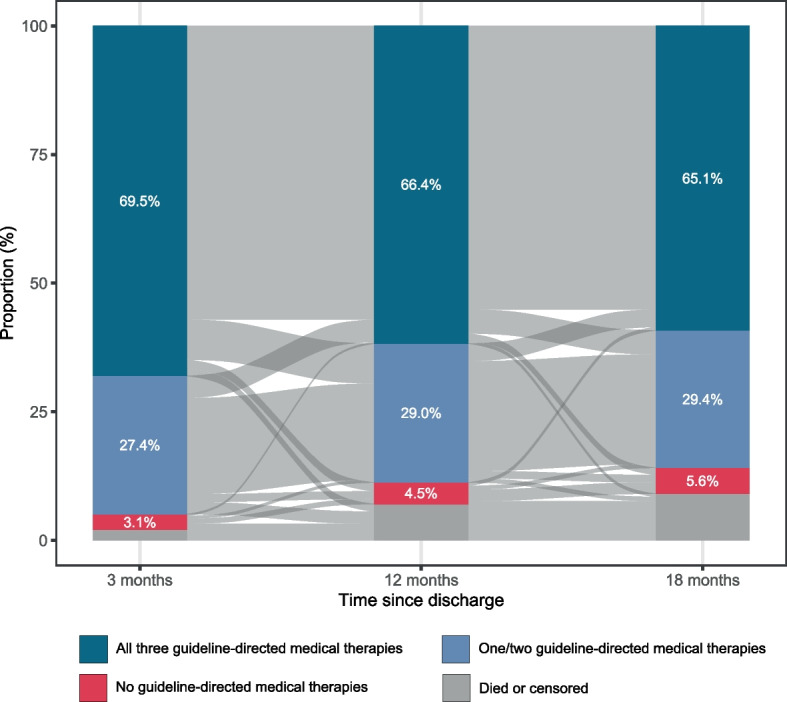
Proportion of patients with myocardial infarction by guideline-directed medical therapy status at 3, 12 and 18 months

The total study population comprised 7,926 patients with incident myocardial infarction (35.4% female, mean age 65 [SD 13] years, 96.8% White, Figure S1). Three months following discharge, 69.5% of patients were receiving all three guideline-directed medical therapies, decreasing to 66.4% at 12 months and 65.1% at 18 months (Fig. 1). Patients who were receiving all three guideline-directed medical therapies at 3 months were younger (62.5 [12.0] *versus* 69.2 [16.8] years), a higher proportion were male (69.2% *versus* 55.8%), and more frequently had one or more comorbidities (Table 1). These patterns were similar at all time points (Table S3-S4). Baseline characteristics for each subgroup are shown in Tables S5-S8*.*

### Provision of guideline-directed medical therapy by subgroups

**Fig. 2:**
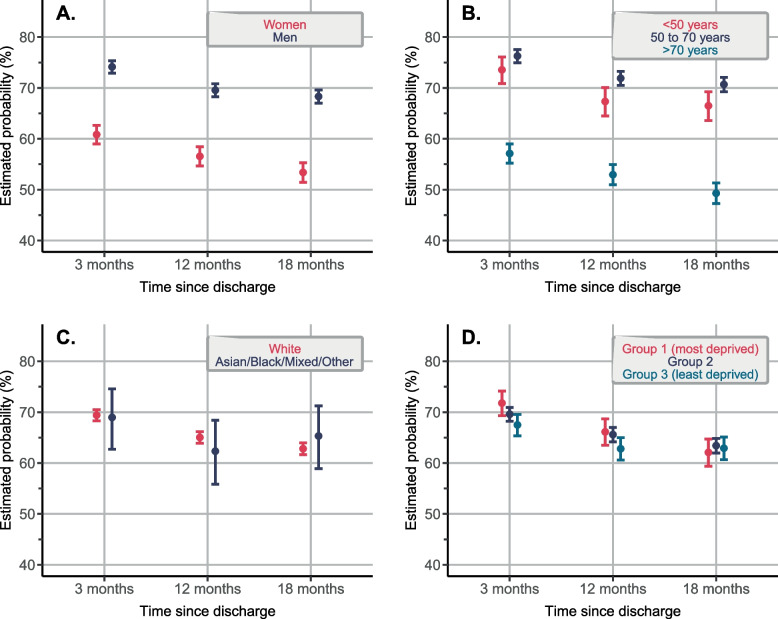
Unadjusted estimated probability of receiving three guideline-directed medical therapies (**A**), (**B**), (**C**), and (**D**) at 3, 12 and 18 months

**Fig. 3:**
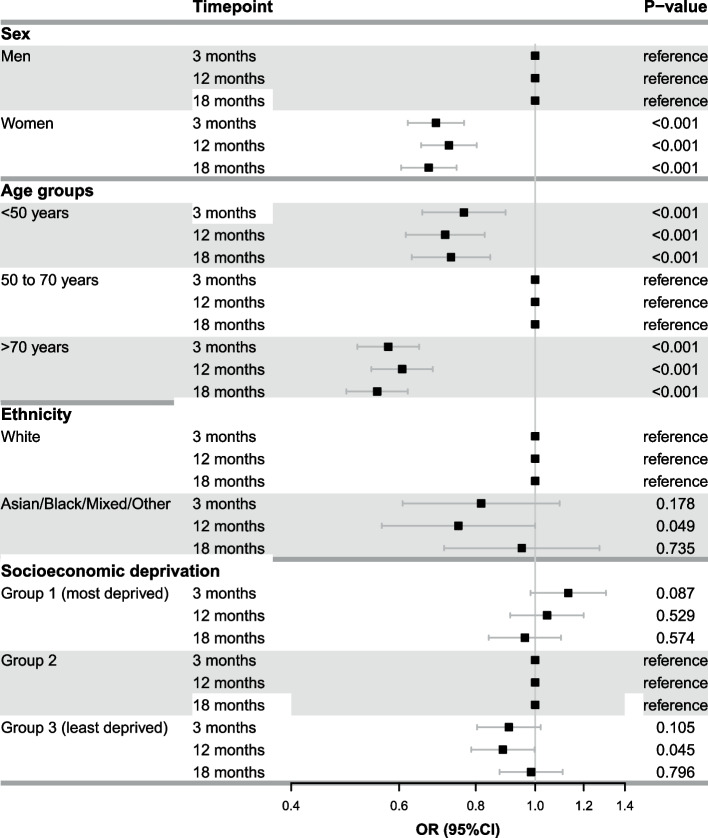
Adjusted odds ratios and their 95% confidence intervals of the association between sociodemographic factors and provision of three guideline-directed medical therapies (*versus* one/two or no therapy, reference category) at 3, 12 and 18 months. Models were adjusted for sex, age, ethnicity, socioeconomic deprivation, and frailty. Abbreviations: OR, odds ratio; CI, confidence interval

**Fig. 4:**
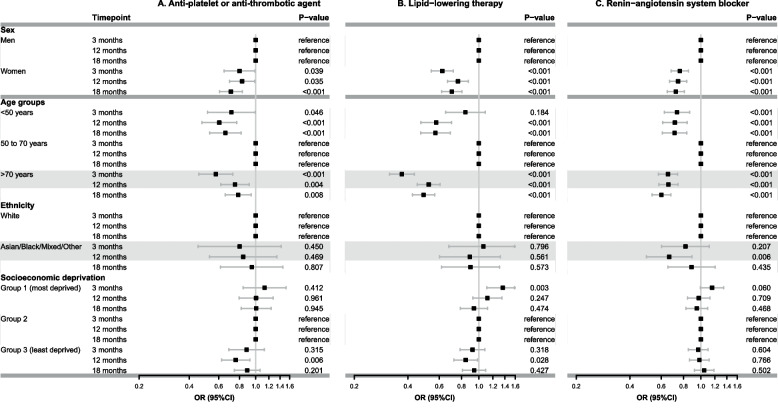
Adjusted odds ratios and their 95% confidence intervals of the association between sociodemographic factors and provision of three guideline-directed medical therapies (*versus* one/two or no therapy, reference category) at 3, 12 and 18 months. Panel **A** Anti-platelet or anti-thrombotic agent; Panel **B** lipid-lowering therapy; Panel **C** renin-angiotensin system blocker. Models were adjusted for sex, age, ethnicity, socioeconomic deprivation, and frailty. Abbreviations: OR, odds ratio; CI, confidence interval

Figure 2 demonstrates the unadjusted estimated probability of receiving all three guideline-directed medical therapies at each time point. In this unadjusted analysis, women and patients > 70 years of age were less likely to receive all three guideline-directed medical therapies as compared with their counterparts at all three time points. These observations persisted in the adjusted analysis (Fig. 3, women versus men, adjusted odds ratio [OR] at 3 months: 0.69 [95% CI 0.62 to 0.77]; and patients > 70 years of age versus 50 to 70 years of age, adjusted OR at 3 months: 0.58 [95% CI 0.51 to 0.65]). The adjusted analysis showed that patients < 50 years of age (as compared to 50 to 70 years of age group) were also less likely to receive guideline-directed medical therapy at all three time points (adjusted OR at 3 months: 0.77 [95% CI 0.65 to 0.89]). No differences were observed across ethnicity and socioeconomic deprivation groups. Similar patterns were found for each of the medications separately (anti-platelet or anti-thrombotic agents, lipid-lowering therapy and RAAS blockers): women and patients < 50 years and > 70 years were less likely to receive all individual medication classes as compared to their counterparts at 3, 12 and 18 months (Fig. 4).

### Guideline-directed medical therapy status and MACE by subgroups

**Fig. 5:**
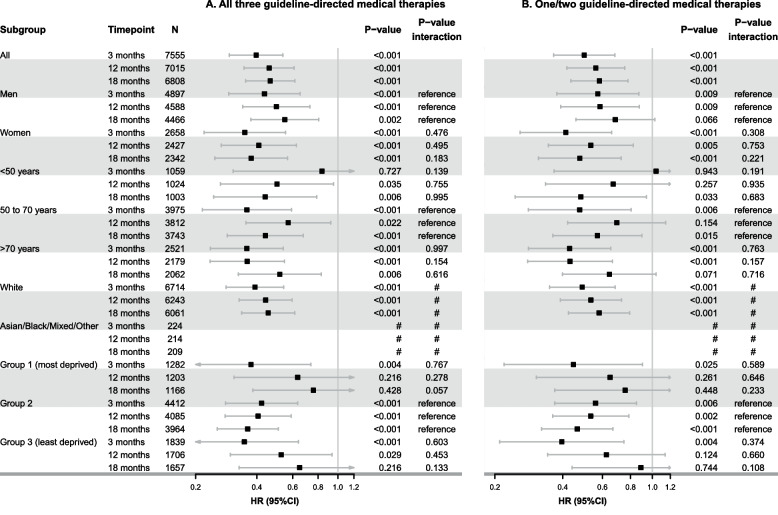
Adjusted hazard ratios and their 95% confidence intervals of the association between guideline-directed medical therapy status and the risk of major adverse cardiac events by sex, age, ethnicity, and socioeconomic deprivation groups. Panel **A** All three guideline-directed medical therapies *versus* none at 3, 12 and 18 months. Panel **B** One/two guideline-directed medical therapy *versus* none at 3, 12 and 18 months. Models were adjusted for sex, age, ethnicity, socioeconomic deprivation, and frailty. # Hazard ratios are not reported for this subgroup due to the limited sample size and the risk of overfitting. Abbreviations: HR, hazard ratio; CI, confidence interval

The crude cumulative incidence of MACE at a median follow-up of 58.4 (25th to 75th percentile, 28.7 to 100.1) months since discharge is shown per subgroup in Table S9. After adjustment, patients receiving either all three guideline-directed medical therapies or one/two guideline-directed medical therapies at 3 months post-discharge had a lower risk of future MACE compared to those who were not receiving any guideline-directed medical therapy (all three medications, adjusted hazard ratio [HR] at 3 months: 0.40 [95% CI 0.29 to 0.54]; and one/two medication(s), adjusted HR at 3 months: 0.50 [95% CI 0.36 to 0.68]). This finding was consistent across all subgroups and landmark time points (Fig. 5).

### Secondary analyses

We conducted several secondary analyses. Investigation of the intersection between sex and age showed that women were less likely to receive guideline-directed medical therapies across all age groups (Figure S2), and both younger and older patients received fewer medications regardless of sex (Figure S3). We found sex differences in relation to socioeconomic deprivation status: women who are living in the least deprived areas were less likely to receive guideline-directed medications compared to other socioeconomic deprivation groups, whereas no such differences were observed among men or across age groups. No sex or age differences were noted in the provision of guideline-directed medical therapy across ethnic groups in our analysis. When stratified by coronary revascularization status, a similar pattern was observed in patients who did and did not undergo revascularization (Figure S4). We divided the cohort into two periods to assess potential temporal differences in the provision of guideline-directed medical therapy. The analysis showed similar results across both periods and were consistent with our primary findings (Figure S5). When we evaluated the co-prescription of four drug classes (anti-platelet or anti-thrombotic agents, lipid-lowering therapy, RAAS blockers, and beta-blockers), patterns in the provision of therapy were consistent with our primary analysis (Figure S6). Moreover, across the study period, we did not observe any notable temporal variation in beta-blocker provision (Figure S7). And finally, we evaluated the impact of comorbidities on the association between guideline-directed medical therapy status and MACE. We found that despite existing comorbidities, receiving either all three guideline-directed medical therapies or one/two guideline-directed medical therapies at 3, 12 and 18 months post-discharge was associated with a lower risk of future MACE compared to patients who were not receiving any guideline-directed medical therapy (Figure S8).

## Discussion

In this study we evaluated trends in the provision of guideline-directed medical therapies following myocardial infarction across sex, age, ethnicity, and socioeconomic deprivation groups. Our study has three main findings. First, only two out of three patients were receiving all three guideline-directed medical therapies at 3 months following myocardial infarction, with a further decrease at 12- and 18-months post-discharge. Second, women, and both younger and older patients were less likely to be receiving guideline-directed medical therapies following myocardial infarction. Finally, patients who received all three or at least one guideline-directed medical therapy had a lower risk of future cardiovascular events compared to those on no guideline-directed medications, with this effect being consistent across all subgroups and time points. Our findings highlight the need for targeted strategies to increase the provision of secondary prevention cardiovascular medical therapies to reduce inequalities and improve post-myocardial infarction care.

Despite the proven effectiveness of secondary prevention medical therapies in reducing the risk of subsequent cardiovascular events after myocardial infarction, uptake is poor worldwide [20]. We found that only two out of three patients are receiving three guideline-directed medical therapies at 3 months with a further decline at 18 months. This is in line with recent data from the United States showing that fewer than half of the patients with known atherosclerotic cardiovascular disease received secondary prevention therapies [21]. Our study provided insights into the subgroups that are particularly prone to underprovision of guideline-directed medical therapy. Future research is urgently needed to identify the drivers of underprovision of evidence-based therapies in these subgroups that may help in the development of tailored interventions to improve the uptake of secondary prevention with the potential to improve outcomes post-myocardial infarction.

Sex- and gender-related disparities have been observed in in-hospital assessment, diagnosis and management of patients with myocardial infarction [22, 23]. In line with previous studies, our study also shows that women are less likely to be receiving guideline-directed medical therapy following a myocardial infarction compared to their male counterparts [4, 5, 9, 11]. Our findings extend current knowledge by showing that this treatment gap persists up to 18 months post-discharge and is observed across all age groups. The underlying mechanisms that are driving these disparities remain unclear and speculative. Women are more likely to be diagnosed with myocardial infarction with non-obstructive coronary arteries (MINOCA) [24], due to spontaneous coronary artery dissection, microvascular disease or Takotsubo cardiomyopathy, which pose diagnostic and therapeutic dilemmas. However, our exploratory analysis found that female patients who had undergone invasive management (i.e., percutaneous coronary intervention or a coronary artery bypass graft) also received fewer secondary prevention medications, suggesting that differences in the pathophysiology of acute coronary syndromes alone do not explain our findings. The impact of differences in patient-caregiver communication between sexes, along with the impact of patient preferences on treatment decisions and on both the prescription and collection of that prescription is still largely unknown. Previous research also suggests that biological differences in drug metabolism may increase women’s risk of adverse side effects from cardiovascular therapies [25] that may alter the subsequent prescription rates of secondary prevention medications in women. As women remain underrepresented in cardiovascular clinical trials [26], our understanding of sex-specific communication, side effects and medication tolerability remains limited. Our outcome analysis showed that the use of guideline-directed medical therapy following myocardial infarction decreased the risk of subsequent cardiovascular events in both female- and male patients, underscoring the importance of identifying the factors that influence the observed disparities in provision of medication by sex.

The persistent underutilization of secondary prevention therapies in patients under 50 years of age, as observed in the current study, aligns with previous research in the United Kingdom showing a higher risk of statin discontinuation in younger patients with myocardial infarction [5]. These findings are concerning as it can be argued that these patients are likely to derive the most benefit from long-term treatment to prevent atherosclerosis. With an ageing population, the number of older patients experiencing a myocardial infarction is expected to rise [27], making the provision of secondary prevention increasingly important to reduce morbidity and mortality in this group. Since our study could not differentiate patients with type 2 myocardial infarction due to non-atherothrombotic mechanisms and the evidence for secondary prevention in this group is lacking, some of the observed differences in use of medication may be related to the higher incidence of type 2 myocardial infarction at extremes of age [28]. Nonetheless, our findings suggest that secondary prevention therapies are currently underutilised in this group. Since older patients may be perceived as more frail and at higher risk of adverse effects of medications, such as postural hypotension, renal injury and bleeding, physicians may not recommend or discontinue preventative treatments more commonly in this group. However, even after accounting for frailty, our findings remained consistent, indicating that frailty alone does not explain the underutilisation of medication in older patients with myocardial infarction. Another reason—although speculative—is that these patients are more likely to have other life limiting illnesses, leading to a perception that secondary prevention therapies offer less benefit in these patients.

In this study, we found no differences in the provision of guideline-directed secondary prevention therapies across ethnic groups. This finding may be due to the limited diversity of our predominantly White population. We acknowledge this as an important limitation of our study and our findings related to ethnicity should be interpreted with caution. A recent study reported that patients of Black and Hispanic ethnicity were less likely to start statin therapy within 30 days of discharge compared to patients who were White, while Asian patients were more likely to do so [29]. Further research evaluating contemporary trends in the provision of secondary prevention therapies across various ethnic groups is needed to ensure optimal post-myocardial infarction care for all ethnic groups. No persistent differences were observed in the provision of secondary prevention medications across socioeconomic deprivation groups in the current study. Given the well-established relationship between ethnicity and socioeconomic status in many health care systems [30], the limited ethnic diversity in our study population may have impacted our findings related to socioeconomic status. Interestingly, when exploring sex-specific prescription patterns, we found that women living in the least deprived areas were less likely to receive guideline-directed medications compared to those in other socioeconomic deprivation groups, while no such differences were observed among men. In contrast, previous research in other countries with universal health care coverage that is free at the point of use, reported that the long-term provision of statin therapy after myocardial infarction was lower among socially deprived individuals, independent of sex [31]. The differences between previous research and our study, may be explained by differences in the definition of socioeconomic deprivation status and/or ethnic diversity. Studies focusing on individual-level socioeconomic deprivation, as well as those exploring the intersection of sex, socioeconomic deprivation, and other relevant factors like age and ethnicity, could provide deeper insights into those at highest risk of under-prescribing and inform targeted interventions to reduce inequalities in cardiovascular care.

Our study has several strengths. First, the use of routinely collected healthcare data enabled us to include a large number of consecutive patients with myocardial infarction who survived to discharge, reducing the risk of bias and allowing insights into current practice. Second, we evaluated the provision of secondary prevention medications up to 18 months. And finally, we evaluated three guideline-directed medications of secondary cardiovascular prevention rather than focusing on lipid-lowering therapy alone. Limitations of our study also need to be acknowledged. First, we evaluated three guideline-recommended medication classes, thereby not accounting for clinical scenarios in which fewer than three medication classes are indicated or where contraindications may exist. Consequently, some patients classified as receiving two or less medications may, in fact, have been appropriately managed based on their individual circumstances. Nonetheless, adjusting for the electronic frailty index did not attenuate our findings, suggesting that observed inequalities in the provision of secondary prevention are not fully explained by differences in frailty or comorbidity burden. Second, our analysis focuses on prescribing data, which does not capture medication dosage or adherence to that prescription. Whilst prescribed medication is an important marker of healthcare provision, the effectiveness of secondary prevention therapies across sociodemographic subgroups depends heavily on dosage and adherence. Differences in adherence across sociodemographic subgroups, as previously observed [32, 33], may attenuate or obscure true associations between guideline-directed medical therapy and subsequent cardiovascular events. Future studies should focus on these elements and incorporating prescription recommendations on discharge from hospital with patient-level adherence data would provide a more comprehensive understanding of the factors influencing treatment gaps. Third, our study utilised electronic health care records, which may have limitations such as potential inaccuracies in coding. Finally, we acknowledge that our MACE outcome analysis may be subject to the influence of potential unmeasured confounding factors such as smoking cessation and participation in cardiac rehabilitation programmes.

In conclusion, women, and both younger and older patients are less likely to be receiving guideline-directed medical therapy between 3 and 18 months following myocardial infarction, despite their known benefit in reducing future cardiovascular events. Targeted strategies to increase the provision of secondary prevention in these groups are needed to reduce inequalities and improve care following myocardial infarction.

## Supplementary Information

Supplementary Material 1.

## Data Availability

The study used routine electronic health care data sources that linked, de-identified and aggregated individual patient level data that is held in a Trusted Research Environment by DataLoch ([https://dataloch.org/](https:/dataloch.org)). The study data and analysis code can be accessed by individuals who have undertaken the necessary governance training on application to DataLoch.

## Abbreviations

aOR: Adjusted Odds Ratio
CI: Confidence Interval
CHI: Community Health Index
HR: Hazard Ratio
MACE: Major Adverse Cardiac Event
MI: Myocardial Infarction
MINOCA: Myocardial Infarction with Non-Obstructive Coronary Arteries
OR: Odds Ratio
ICD-10: International Classification of Diseases 10
RAAS: Renin-Angiotensin Aldosterone System
SD: Standard Deviation
SIMD: Scottish Index of Multiple Deprivation
