# Supplementary material for: Inequalities in the provision of guideline-directed medical therapy following myocardial infarction: a cohort study
Source: BMC Cardiovasc Disord. 2026 Feb 6;26:213. doi: 10.1186/s12872-026-05572-8 (PMC12977493; doi:10.1186/s12872-026-05572-8)
Supplement: Supplementary file 1 — Supplementary Material 1. [file 12872_2026_5572_MOESM1_ESM.docx]

SUPPLEMENTARY APPENDIX

**Inequalities in the provision of guideline-directed medical therapy following myocardial infarction: a cohort study**

**Running title:** Inequalities in therapy following MI

Fiona McLachlan*^1^, Marie de Bakker*^1^, Cesario Pancinha^1^, Thomas M Caparrotta^2,3^, Caroline Jackson^1^, Thulani Ashcroft^2^, Atul Anand^2^, Peter J Gallacher^2^, Eve Miller-Hodges^2,4^, David Yeung^2^, Neeraj Dhaun^2,4^, Chris Tuck^2^, Nicholas L Mills^1,2^, Dorien M Kimenai^1^

*^1^Usher Institute, University of Edinburgh, Edinburgh, United Kingdom*

*^2^British Heart Foundation Centre for Cardiovascular Science, University of Edinburgh, United Kingdom*

*^3^Clinical Pharmacology Unit and Research Centre, University of Edinburgh, United Kingdom*

*^4^Department of Renal Medicine, Royal Infirmary of Edinburgh, Edinburgh, United Kingdom*

*Contributed equally

**Address for correspondence:**

Dorien Kimenai, PhD

Usher Institute

The University of Edinburgh

Usher Building, Edinburgh EH16 4UX United Kingdom

Email: [dorien.kimenai@ed.ac.uk](mailto:dorien.kimenai@ed.ac.uk)

Twitter: @DorienKimenai

**Figures and Tables**

**Figure S1. Study flow diagram.**

**Figure S2.** **Age-specific adjusted odds ratio and their 95% confidence intervals of the association between sociodemographic factors and provision of three guideline-directed medical therapies (*versus* one/two or no therapy, reference category) at 3, 12 and 18 months.** Panel A: <50 years; Panel B: 50 to 70 years; Panel C: >70 years. Models were adjusted for sex, ethnicity, socioeconomic deprivation, and frailty. Abbreviations: OR, odds ratio; CI, confidence interval.

**Figure S3. Sex-specific adjusted odds ratio and their 95% confidence intervals of the association between sociodemographic factors and provision of three guideline-directed medical therapies (*versus* one/two or no therapy, reference category) at 3, 12 and 18 months.** Panel A: Women; Panel B: Men. Models were for adjusted age, ethnicity, socioeconomic deprivation, and frailty. Abbreviations: OR, odds ratio; CI, confidence interval.

**Figure S4. Adjusted odds ratio and their 95% confidence intervals of the association between sociodemographic factors and provision of three guideline-directed medical therapies (versus one/two or no therapy, reference category) at 3, 12 and 18 months.** Panel A: patients who received coronary revascularisation; Panel B: patients who didn’t receive revascularisation. Models were adjusted for sex, age, ethnicity, socioeconomic deprivation, and frailty. Abbreviations: OR, odds ratio; CI, confidence interval.

**Figure S5. Adjusted odds ratios and their 95% confidence intervals of the association between sociodemographic factors and provision of three guideline-directed medical therapies (versus one/two or no therapy, reference category) at 3, 12 and 18 months by time period.** Models were adjusted for sex, age, ethnicity, socioeconomic deprivation, and frailty. Abbreviations: OR, odds ratio; CI, confidence interval. Panel A: Time period: 1st of April 2009 - 31st of March 2015; Panel B: Time period: 1st of April 2015 – 31st of July 2021.

**Figure S6. Adjusted odds ratios and their 95% confidence intervals of the association between sociodemographic factors and provision of three guideline-directed medical therapies included beta blockers (*versus* one/two or no therapy, reference category) at 3, 12 and 18 months.** Models were for adjusted sex, age, ethnicity, socioeconomic deprivation, and frailty. Abbreviations: OR, odds ratio; CI, confidence interval.

**Figure S7. The proportion of patients receiving beta-blocker therapy at 3 months post-discharge, stratified by the year of their incident myocardial infarction.**

**Figure S8. Adjusted hazard ratios and their 95% confidence intervals of the association between guideline-directed medical therapy status and the risk of major adverse cardiac events by sex, age, ethnicity, and socioeconomic deprivation groups.** Panel A: All three guideline-directed medical therapies versus none at 3, 12 and 18 months. Panel B: One/two guideline-directed medical therapy versus none at 3, 12 and 18 months. Models were adjusted for sex, age, ethnicity, socioeconomic deprivation, renal function, and history of diabetes, heart failure, hypertension, obesity, stroke, or transient ischemic attack. # Hazard ratios are not reported for this subgroup due to the limited sample size and the risk of overfitting. Abbreviations: HR, hazard ratio; CI, confidence interval.

**Table S1. The RECORD checklist – extended from the STROBE statement.**

**Table S2. Included prescribed medications.**

**Table S3. Baseline characteristics of patients with myocardial infarction by guideline-medical therapy status at 12 months.**

**Table S4.** **Baseline characteristics of patients with myocardial infarction by guideline-medical therapy status at 18 months.**

**Table S5. Baseline characteristics of patients with myocardial infarction by sex.**

**Table S6. Baseline characteristics of patients with myocardial infarction by age into <50 years, 50 to 70 years, and >70 years groups.**

**Table S7. Baseline characteristics of patients with myocardial infarction by ethnicity into White and Asian/Black/Mixed/Other groups.**

**Table S8. Baseline characteristics of patients with myocardial infarction by socioeconomic deprivation status into group 1 (most deprived), group 2 and group 3 (least deprived).**

**Table S9. Crude event rates by sex, ethnicity, and socioeconomic deprivation groups.** Median follow-up was 58.4 [25^th^ percentile, 75^th^ percentile, 28.7, 100.1] months.

**
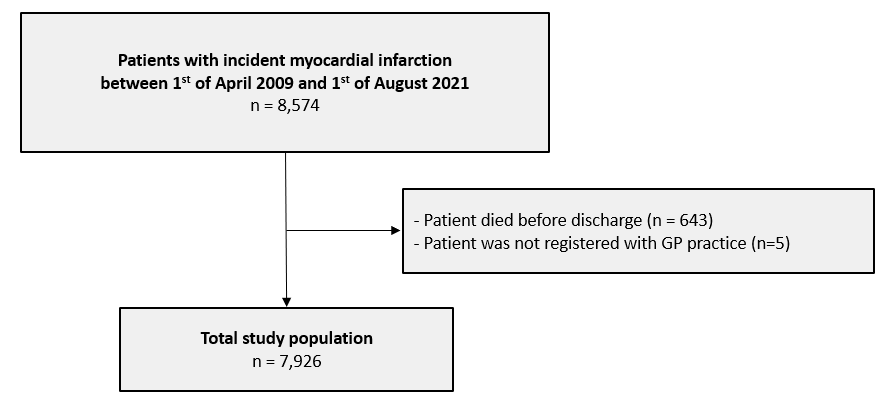
**

**Figure S1. Study flow diagram.**

**
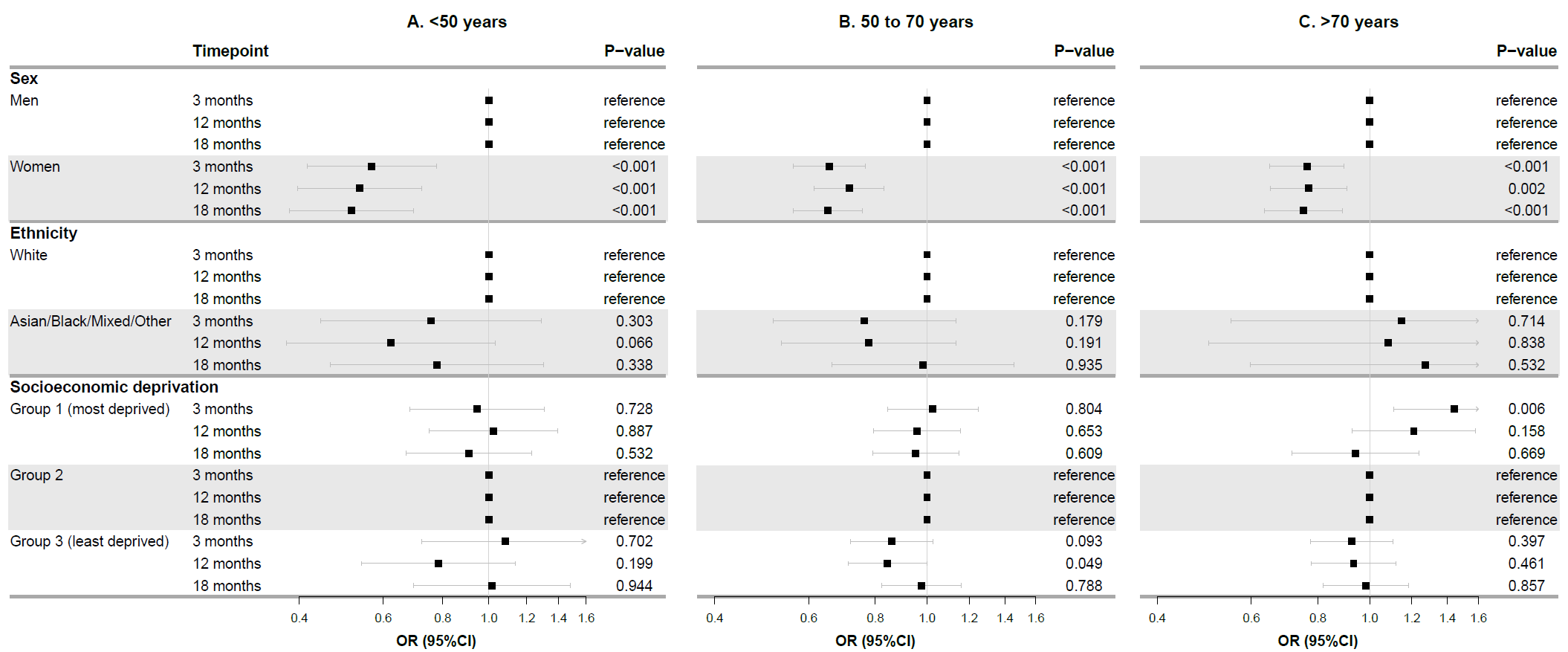
**

**Figure S2.** **Age-specific adjusted odds ratio and their 95% confidence intervals of the association between sociodemographic factors and provision of three guideline-directed medical therapies (*versus* one/two or no therapy, reference category) at 3, 12 and 18 months.** Panel A: <50 years; Panel B: 50 to 70 years; Panel C: >70 years. Models were adjusted for sex, ethnicity, socioeconomic deprivation, and frailty. Abbreviations: OR, odds ratio; CI, confidence interval.

**
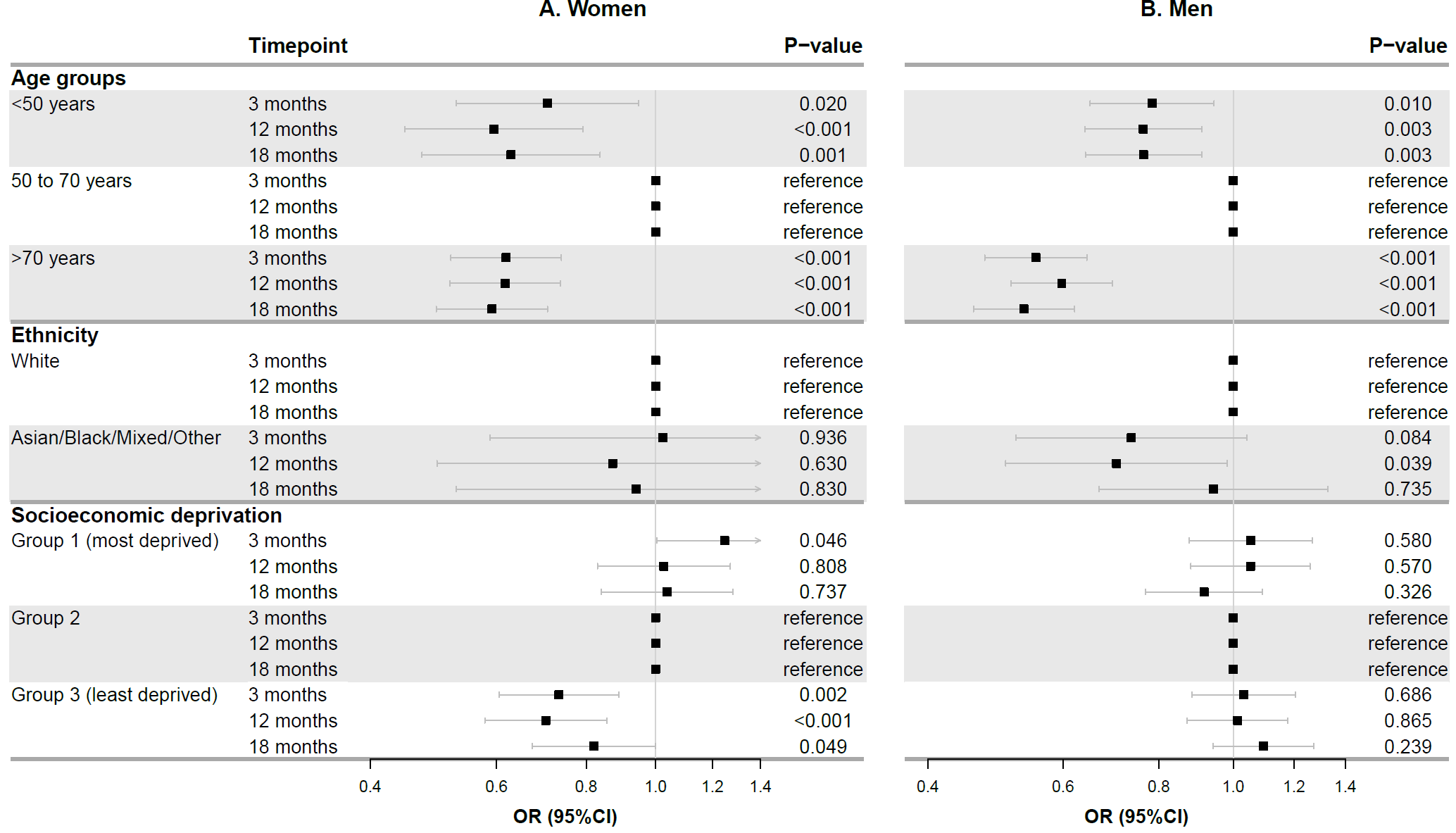
**

**Figure S3. Sex-specific adjusted odds ratio and their 95% confidence intervals of the association between sociodemographic factors and provision of all guideline-directed medical therapies (*versus* one/two or no therapy, reference category) at 3, 12 and 18 months.** Panel A: Women; Panel B: Men. Models were for adjusted age, ethnicity, socioeconomic deprivation, and frailty. Abbreviations: OR, odds ratio; CI, confidence interval.


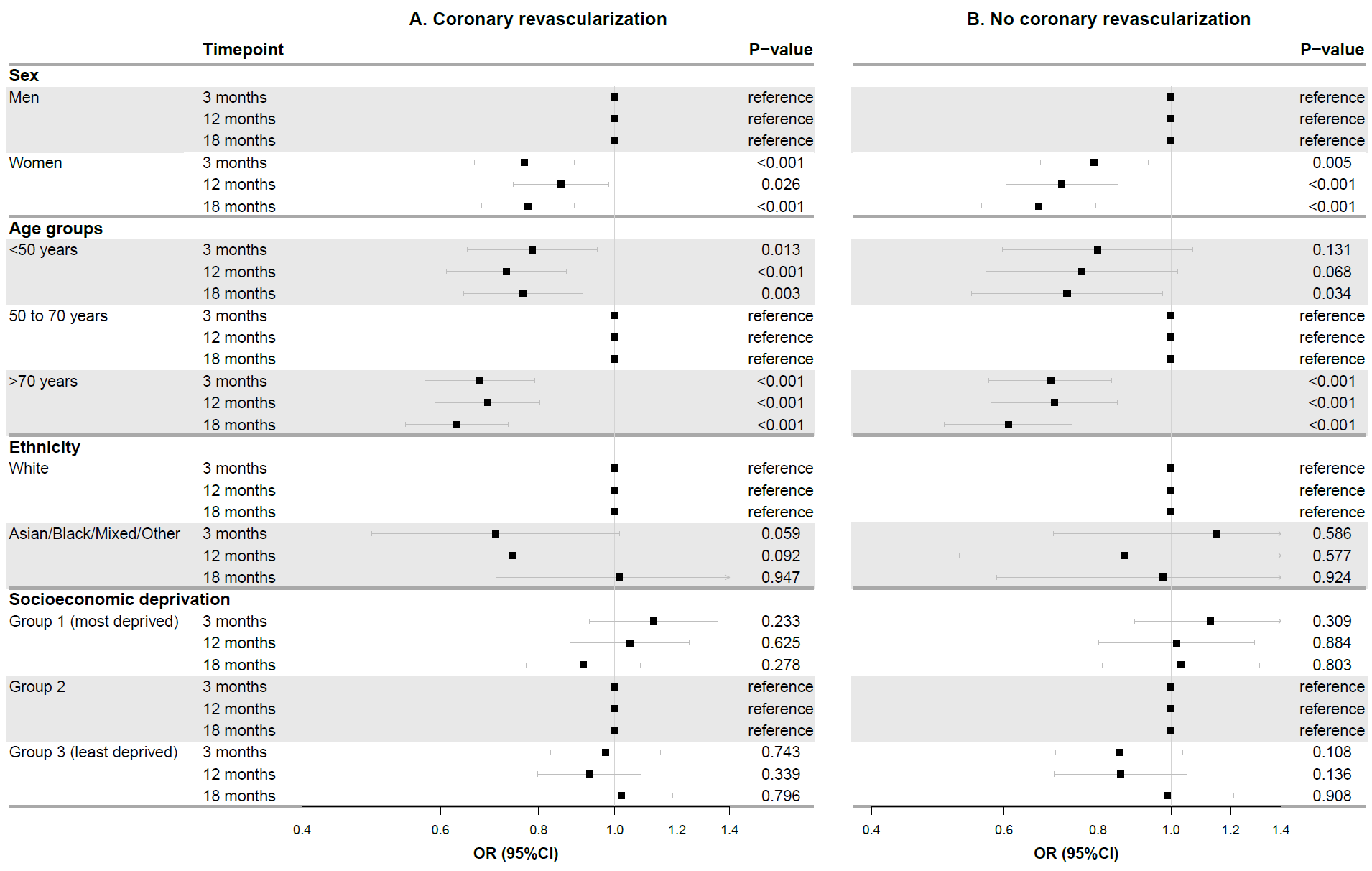


**Figure S4.** **Adjusted odds ratio and their 95% confidence intervals of the association between sociodemographic factors and provision of all guideline-directed medical therapies (*versus* one/two or no therapy, reference category) at 3, 12 and 18 months.** Panel A: patients who received coronary revascularisation; Panel B: patients who didn’t receive revascularisation. Models were adjusted for sex, age, ethnicity, socioeconomic deprivation, and frailty. Abbreviations: OR, odds ratio; CI, confidence interval.


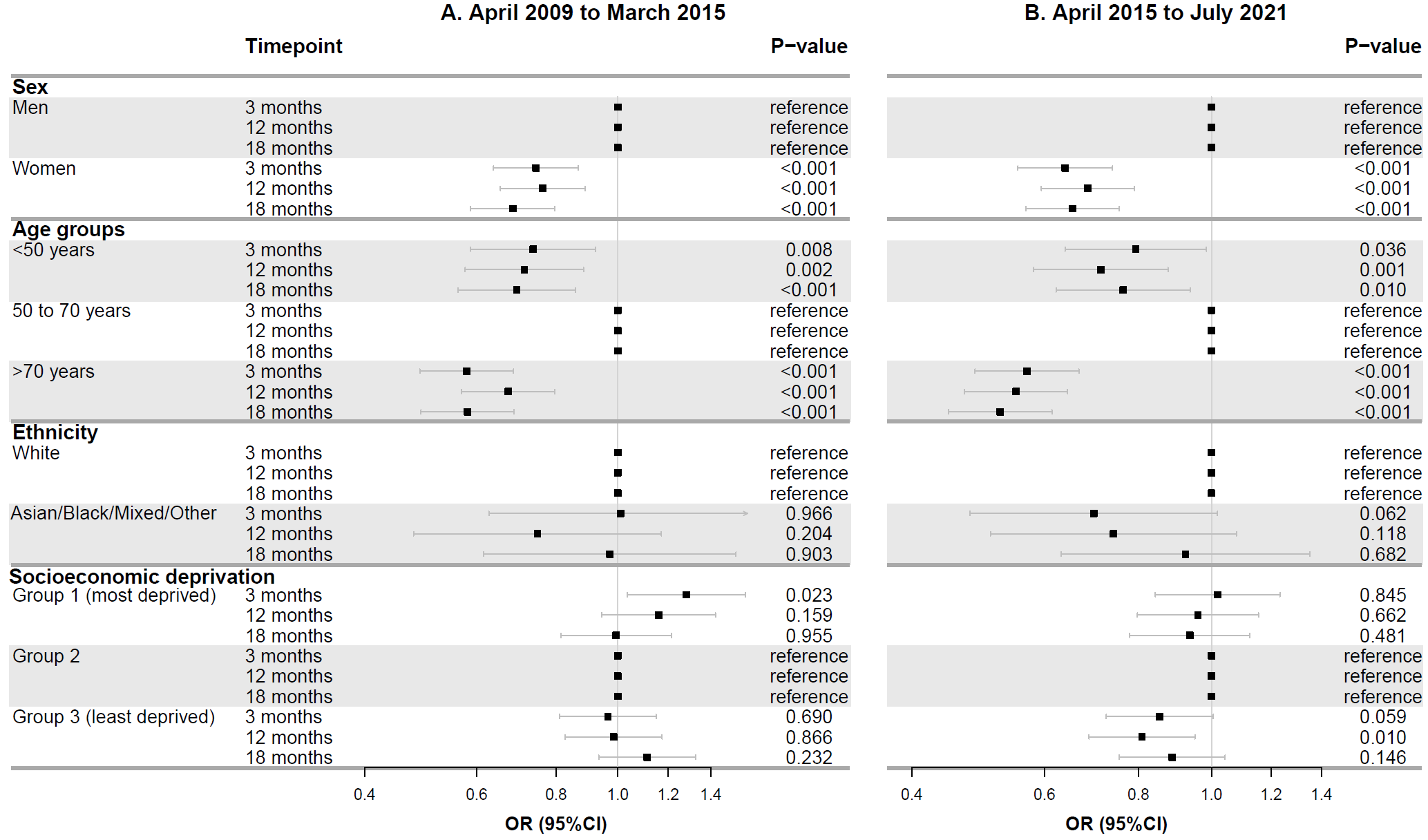


**Supplemental Figure S5. Adjusted odds ratios and their 95% confidence intervals of the association between sociodemographic factors and provision of three guideline-directed medical therapies (versus one/two or no therapy, reference category) at 3, 12 and 18 months by time period**. Models were adjusted for sex, age, ethnicity, socioeconomic deprivation, and frailty. Abbreviations: OR, odds ratio; CI, confidence interval. Panel A: Time period: 1st of April 2009 - 31st of March 2015; Panel B: Time period: 1st of April 2015 – 31st of July 2021.

**
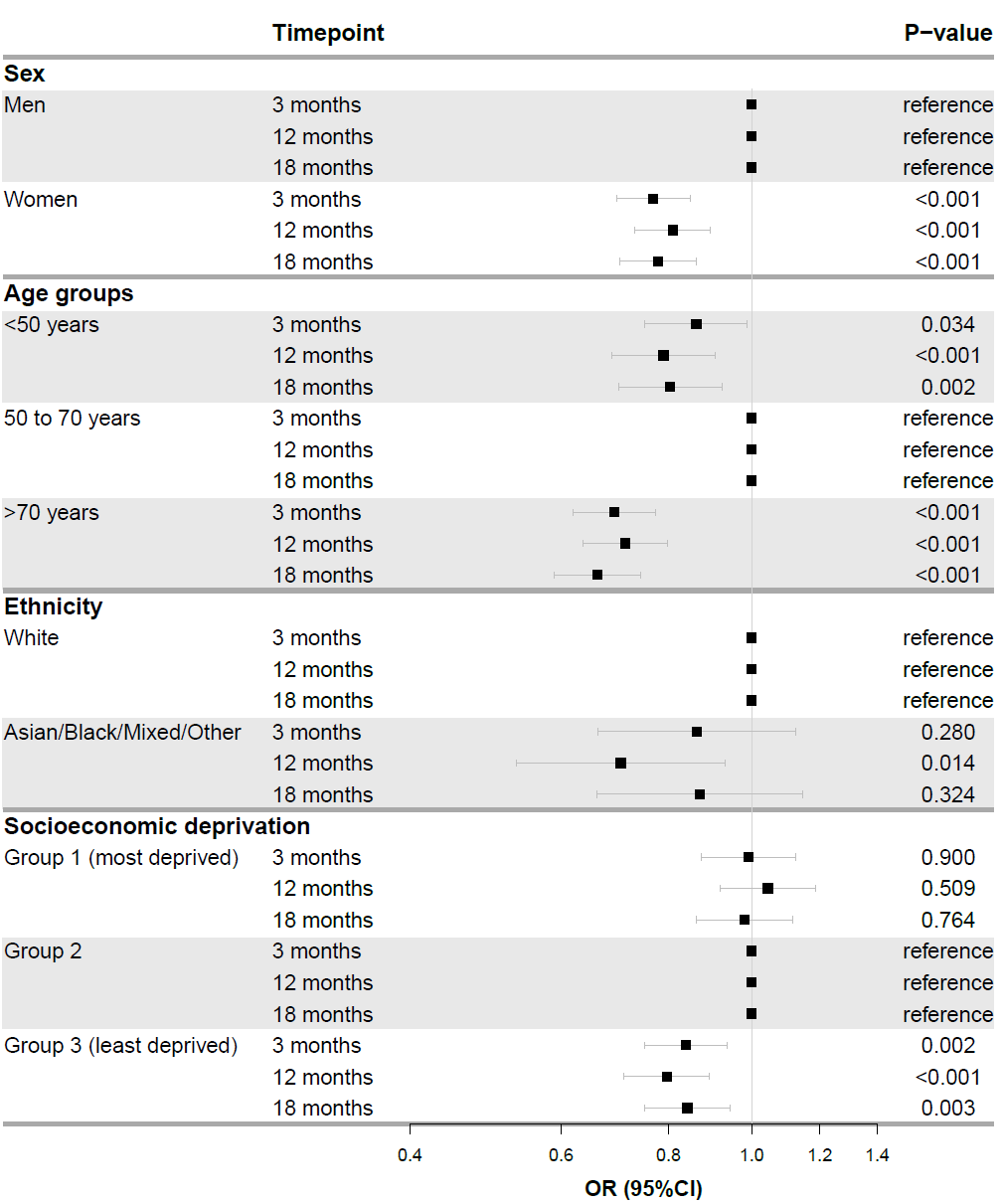
**

**Figure S6. Adjusted odds ratios and their 95% confidence intervals of the association between sociodemographic factors and provision of all guideline-directed medical therapies included beta blockers (*versus* one/two or no therapy, reference category) at 3, 12 and 18 months.** Models were for adjusted sex, age, ethnicity, socioeconomic deprivation, and frailty. Abbreviations: OR, odds ratio; CI, confidence interval.


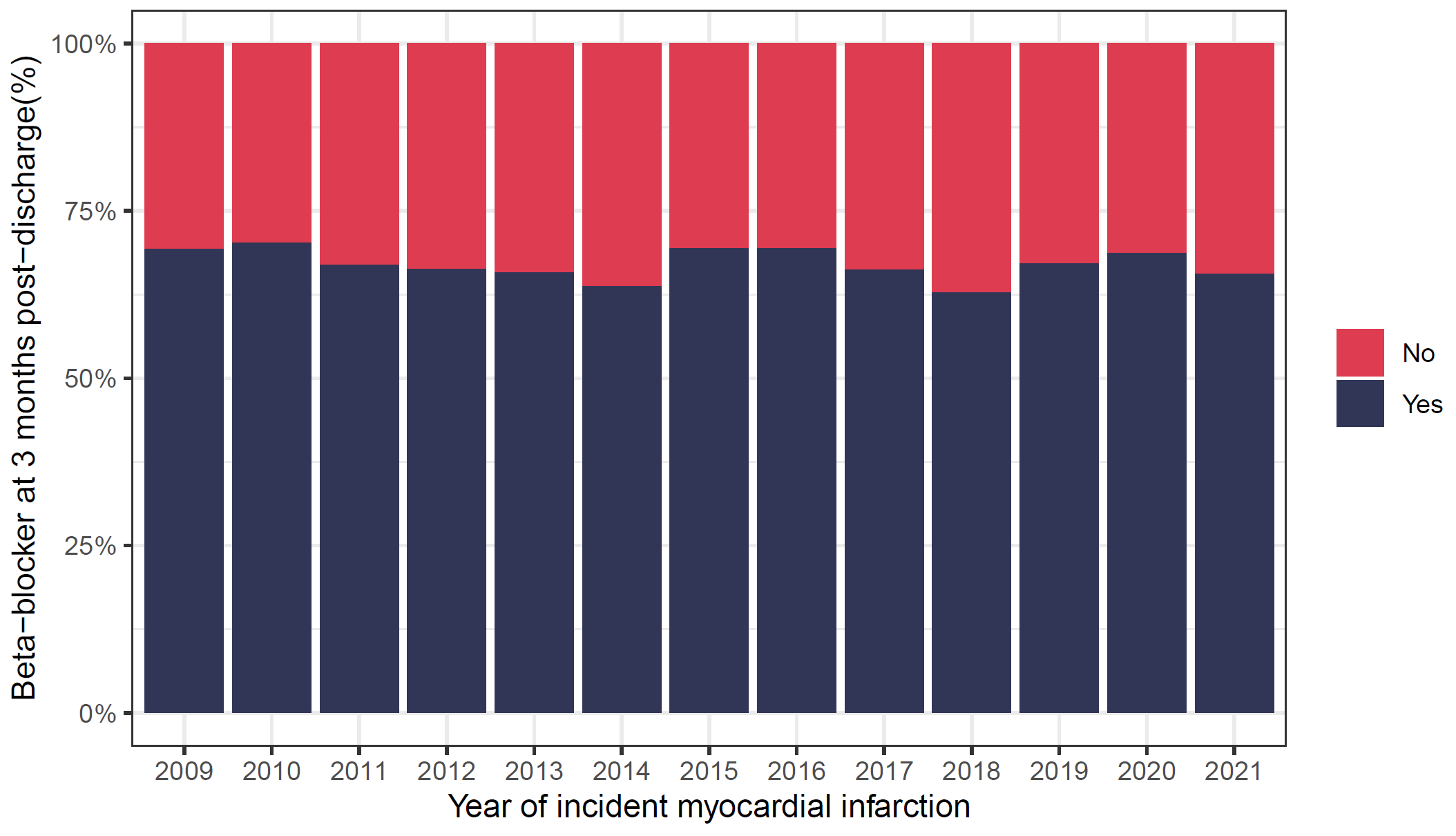


**Figure S7. The proportion of patients receiving beta-blocker therapy at 3 months post-discharge, stratified by the year of their incident myocardial infarction.**


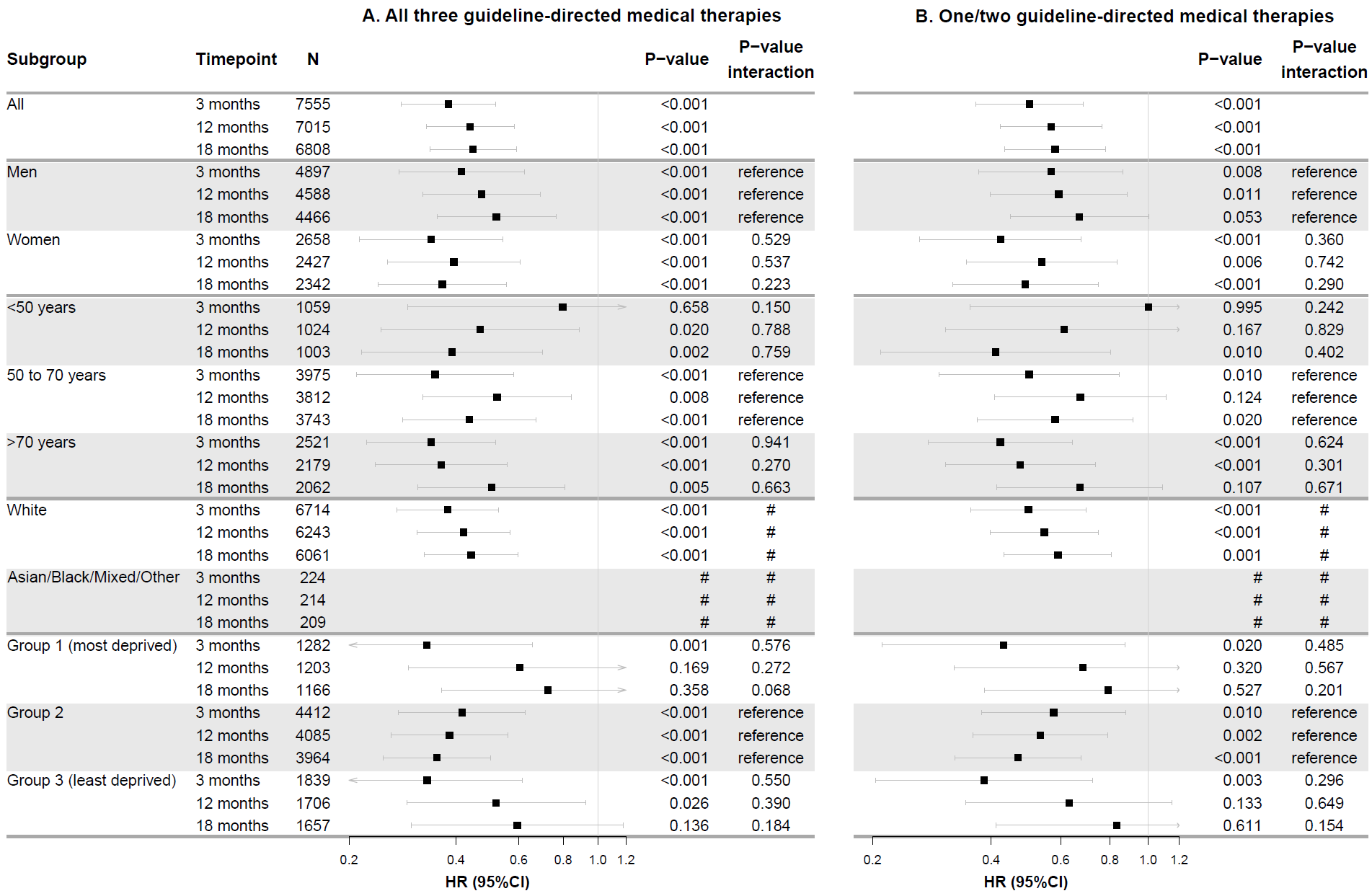


**Supplemental Figure S8. Adjusted hazard ratios and their 95% confidence intervals of the association between guideline-directed medical therapy status and the risk of major adverse cardiac events by sex, age, ethnicity, and socioeconomic deprivation groups.** Panel A: All three guideline-directed medical therapies versus none at 3, 12 and 18 months. Panel B: One/two guideline-directed medical therapy versus none at 3, 12 and 18 months. Models were adjusted for sex, age, ethnicity, socioeconomic deprivation, renal function, and history of diabetes, heart failure, hypertension, obesity, stroke, or transient ischemic attack. # Hazard ratios are not reported for this subgroup due to the limited sample size and the risk of overfitting. Abbreviations: HR, hazard ratio; CI, confidence interval.

**Table S1. The RECORD checklist – extended from the STROBE statement.**

|  | **Item No.** | **STROBE items** | **Location in manuscript where items are reported** | **RECORD items** | **Location in manuscript where items are reported** |
| --- | --- | --- | --- | --- | --- |
| **Title and abstract** | | | | | |
|  | 1 | (a) Indicate the study’s design with a commonly used term in the title or the abstract (b) Provide in the abstract an informative and balanced summary of what was done and what was found | Page 1, lines 4  Page 2, lines 6-7 | RECORD 1.1: The type of data used should be specified in the title or abstract. When possible, the name of the databases used should be included.  RECORD 1.2: If applicable, the geographic region and timeframe within which the study took place should be reported in the title or abstract.  RECORD 1.3: If linkage between databases was conducted for the study, this should be clearly stated in the title or abstract. | Page 2, lines 6-7  Page 2, lines 6-7  Page 2, lines 6-7 |
| **Introduction** | | | | | |
| Background rationale | 2 | Explain the scientific background and rationale for the investigation being reported | Page 3, lines 2-20 |  |  |
| Objectives | 3 | State specific objectives, including any prespecified hypotheses | Page 3, lines 22-24  Page 4, lines 1-2 |  |  |
| **Methods** | | | | | |
| Study Design | 4 | Present key elements of study design early in the paper | Page 4, lines 5-21 |  |  |
| Setting | 5 | Describe the setting, locations, and relevant dates, including periods of recruitment, exposure, follow-up, and data collection | Page 4-6 |  |  |
| Participants | 6 | *(a) Cohort study* - Give the eligibility criteria, and the sources and methods of selection of participants. Describe methods of follow-up  *Case-control study* - Give the eligibility criteria, and the sources and methods of case ascertainment and control selection. Give the rationale for the choice of cases and controls  *Cross-sectional study* - Give the eligibility criteria, and the sources and methods of selection of participants  *(b) Cohort study* - For matched studies, give matching criteria and number of exposed and unexposed  *Case-control study* - For matched studies, give matching criteria and the number of controls per case | Page 4, lines 5-21  Page 5, lines 1-12 | RECORD 6.1: The methods of study population selection (such as codes or algorithms used to identify subjects) should be listed in detail. If this is not possible, an explanation should be provided.  RECORD 6.2: Any validation studies of the codes or algorithms used to select the population should be referenced. If validation was conducted for this study and not published elsewhere, detailed methods and results should be provided.  RECORD 6.3: If the study involved linkage of databases, consider use of a flow diagram or other graphical display to demonstrate the data linkage process, including the number of individuals with linked data at each stage. | Page 4, lines 5-21  Page 4, lines 5-21  Page 5, lines 1-12 |
| Variables | 7 | Clearly define all outcomes, exposures, predictors, potential confounders, and effect modifiers. Give diagnostic criteria, if applicable. | Page 5, lines 14-25  Page 6, lines 1-20 | RECORD 7.1: A complete list of codes and algorithms used to classify exposures, outcomes, confounders, and effect modifiers should be provided. If these cannot be reported, an explanation should be provided. | Page 5, lines 14-25  Page 6, lines 1-20 |
| Data sources/ measurement | 8 | For each variable of interest, give sources of data and details of methods of assessment (measurement).  Describe comparability of assessment methods if there is more than one group | Page 5, lines 1-12 |  |  |
| Bias | 9 | Describe any efforts to address potential sources of bias | Page 4-6 |  |  |
| Study size | 10 | Explain how the study size was arrived at | Page 4, lines 5-21 |  |  |
| Quantitative variables | 11 | Explain how quantitative variables were handled in the analyses. If applicable, describe which groupings were chosen, and why | Page 6-8 |  |  |
| Statistical methods | 12 | (a) Describe all statistical methods, including those used to control for confounding  (b) Describe any methods used to examine subgroups and interactions  (c) Explain how missing data were addressed  (d) *Cohort study* - If applicable, explain how loss to follow-up was addressed  *Case-control study* - If applicable, explain how matching of cases and controls was addressed  *Cross-sectional study* - If applicable, describe analytical methods taking account of sampling strategy  (e) Describe any sensitivity analyses | Page 6, lines 22-24  Page 7, lines 1-25  Page 8, lines 2-9 |  |  |
| Data access and cleaning methods |  | .. |  | RECORD 12.1: Authors should describe the extent to which the investigators had access to the database population used to create the study population.  RECORD 12.2: Authors should provide information on the data cleaning methods used in the study. | Page 4, lines 5-21  Page 4, lines 5-21 |
| Linkage |  | .. |  | RECORD 12.3: State whether the study included person-level, institutional-level, or other data linkage across two or more databases. The methods of linkage and methods of linkage quality evaluation should be provided. | Page 4, lines 5-21 |
| **Results** | | | | | |
| Participants | 13 | (a) Report the numbers of individuals at each stage of the study (*e.g.*, numbers potentially eligible, examined for eligibility, confirmed eligible, included in the study, completing follow-up, and analysed)  (b) Give reasons for non-participation at each stage.  (c) Consider use of a flow diagram | Figure S1 | RECORD 13.1: Describe in detail the selection of the persons included in the study (*i.e.,* study population selection) including filtering based on data quality, data availability and linkage. The selection of included persons can be described in the text and/or by means of the study flow diagram. | Figure S1 |
| Descriptive data | 14 | (a) Give characteristics of study participants (*e.g.*, demographic, clinical, social) and information on exposures and potential confounders  (b) Indicate the number of participants with missing data for each variable of interest  (c) *Cohort study* - summarise follow-up time (*e.g.*, average and total amount) | Table 1  Page 8, lines 16-24  Page 9, lines 1-3 |  |  |
| Outcome data | 15 | *Cohort study* - Report numbers of outcome events or summary measures over time  *Case-control study* - Report numbers in each exposure category, or summary measures of exposure  *Cross-sectional study* - Report numbers of outcome events or summary measures | Page 9, lines 5-24  Page 10, lines 1-22 |  |  |
| Main results | 16 | (a) Give unadjusted estimates and, if applicable, confounder-adjusted estimates and their precision (e.g., 95% confidence interval). Make clear which confounders were adjusted for and why they were included  (b) Report category boundaries when continuous variables were categorized  (c) If relevant, consider translating estimates of relative risk into absolute risk for a meaningful time period | Page 9, lines 5-24  Page 10, lines 1-6 |  |  |
| Other analyses | 17 | Report other analyses done—e.g., analyses of subgroups and interactions, and sensitivity analyses | Page 10, lines 8-22 |  |  |
| **Discussion** | | | | | |
| Key results | 18 | Summarise key results with reference to study objectives | Page 11, lines 2-13 |  |  |
| Limitations | 19 | Discuss limitations of the study, taking into account sources of potential bias or imprecision. Discuss both direction and magnitude of any potential bias | Page 14, lines 25-26  Page 15, lines 1-11 | RECORD 19.1: Discuss the implications of using data that were not created or collected to answer the specific research question(s). Include discussion of misclassification bias, unmeasured confounding, missing data, and changing eligibility over time, as they pertain to the study being reported. | Page 14, lines 25-26  Page 15, lines 1-11 |
| Interpretation | 20 | Give a cautious overall interpretation of results considering objectives, limitations, multiplicity of analyses, results from similar studies, and other relevant evidence | Page 11-15 |  |  |
| Generalisability | 21 | Discuss the generalisability (external validity) of the study results | Page 11-15 |  |  |
| **Other Information** | | | | | |
| Funding | 22 | Give the source of funding and the role of the funders for the present study and, if applicable, for the original study on which the present article is based | Page 18, lines 2-11 |  |  |
| Accessibility of protocol, raw data, and programming code |  | .. |  | RECORD 22.1: Authors should provide information on how to access any supplemental information such as the study protocol, raw data, or programming code. | Page 17, lines 8-13 |

**Table S2. Included prescribed medications.**

| **Anti-platelet or anti-thrombotic agent** | **Lipid-lowering therapy** | **Renin-angiotensin aldosterone blockers** | **Betablockers** |
| --- | --- | --- | --- |
| acenocoumarol | alirocumab | candesartan cilexetil | acebutolol hydrochloride |
| apixaban | atorvastatin | captopril | atenolol |
| aspirin | bempedoic acid | co-zidocapt (hydchloroth/captopril) | atenolol with calcium channel blocker |
| clopidogrel | bempedoic acid/ezetimibe | enalapril maleate | bisoprolol fumarate |
| dabigatran etexilate | bezafibrate | enalapril maleate with diuretic | carvedilol |
| dalteparin sodium | evolocumab | eprosartan | celiprolol hydrochloride |
| dipyridamole | ezetimibe | fosinopril sodium | co-tenidone (atenolol/chlortalidone |
| edoxaban | fenofibrate | imidapril hydrochloride | labetalol hydrochloride |
| enoxaparin | fluvastatin sodium | irbesartan | metoprolol tartrate |
| fondaparinux sodium | gemfibrozil | irbesartan with diuretic | nebivolol |
| heparin sodium | pravastatin sodium | lisinopril | propranolol hydrochloride |
| prasugrel | rosuvastatin calcium | lisinopril with diuretic | sotalol hydrochloride |
| rivaroxaban | simvastatin | losartan potassium | timolol |
| ticagrelor | simvastatin & ezetimibe | losartan potassium with diuretic |  |
| tinzaparin sodium | simvastatin and ezetimibe | olmesartan medoxomil |  |
| warfarin sodium |  | perindopril arginine |  |
|  |  | perindopril arginine with diuretic |  |
|  |  | perindopril erbumine |  |
|  |  | quinapril hydrochloride |  |
|  |  | quinapril hydrochloride with diuretic |  |
|  |  | ramipril |  |
|  |  | sacubitril/valsartan |  |
|  |  | telmisartan |  |
|  |  | telmisartan with diuretic |  |
|  |  | trandolapril |  |
|  |  | valsartan |  |
|  |  | valsartan with diuretic |  |
|  |  | valsartan/amlodipine |  |

**Table S3. Baseline characteristics of patients with myocardial infarction by guideline-medical therapy status at 12 months.**

|  | **Guideline-directed medical therapy at 12 months** | | | |
| --- | --- | --- | --- | --- |
| **Characteristic** | **Three n = 4895** | **One/two n = 2139** | **None n = 333** | **Died or censored n = 559** |
| Age (years) | 62.3 (11.7) | 67.0 (14.2) | 63.3 (16.4) | 75.2 (14.3) |
| Sex (% male) | 3,401 (69.5%) | 1,207 (56.4%) | 196 (58.9%) | 320 (57.2%) |
| Ethnicity (%) |  |  |  |  |
| White | 4,358 (96.8%) | 1,918 (96.6%) | 284 (95.0%) | 473 (98.1%) |
| Asian/Black/Mixed/Other | 143 (3.2%) | 68 (3.4%) | 15 (5.0%) | 9 (1.9%) |
| Socioeconomic deprivation* (%) |  |  |  |  |
| Group 1 (most deprived) | 847 (17.3%) | 355 (16.6%) | 60 (18.2%) | 92 (16.5%) |
| Group 2 | 2,883 (59.0%) | 1,229 (57.6%) | 179 (54.4%) | 329 (59.1%) |
| Group 3 (least deprived) | 1,153 (23.6%) | 550 (25.8%) | 90 (27.4%) | 136 (24.4%) |
| History of diabetes (%) | 713 (14.6%) | 338 (15.8%) | 48 (14.4%) | 139 (24.9%) |
| History of hypertension (%) | 1,939 (39.6%) | 877 (41.0%) | 111 (33.3%) | 347 (62.1%) |
| History of obesity (%) | 926 (18.9%) | 388 (18.1%) | 51 (15.3%) | 112 (20.0%) |
| History of stroke and/or TIA (%) | 256 (5.2%) | 200 (9.4%) | 24 (7.2%) | 111 (19.9%) |
| History of heart failure (%) | 101 (2.1%) | 105 (4.9%) | 16 (4.8%) | 89 (15.9%) |
| Current smoker (%) | 1,650 (39.4%) | 643 (34.1%) | 116 (41.4%) | 130 (25.7%) |
| Body mass index (kg/m2) | 28.6 [25.5, 32.3] | 27.0 [23.7, 30.8] | 26.2 [22.4, 30.4] | 25.8 [22.6, 29.9] |
| Systolic blood pressure (mmHg) | 137.5 (17.5) | 136.1 (18.5) | 135.9 (19.9) | 136.1 (20.3) |
| Diastolic blood pressure (mmHg) | 80.5 (11.1) | 78.1 (11.4) | 79.3 (11.6) | 76.0 (12.1) |
| Estimated glomerular filtration rate (% yes) |  |  |  |  |
| <30 ml/min/1.73m2 | 32 (0.7%) | 81 (3.9%) | 9 (2.8%) | 66 (12.0%) |
| 30 to 44 ml/min/1.73m2 | 123 (2.5%) | 135 (6.4%) | 21 (6.5%) | 79 (14.4%) |
| 45 to 59 ml/min/1.73m2 | 335 (6.9%) | 215 (10.2%) | 22 (6.8%) | 104 (18.9%) |
| ≥ 60 ml/min/1.73m2 | 4,353 (89.9%) | 1,670 (79.5%) | 272 (84.0%) | 300 (54.6%) |
| Cholesterol (mmol/L) | 5.2 (1.3) | 5.1 (1.3) | 5.2 (1.3) | 4.6 (1.3) |
| Low-density lipoprotein cholesterol (mmol/L) | 3.2 (1.1) | 3.2 (1.1) | 3.3 (1.1) | 2.8 (1.1) |
| High-density lipoprotein cholesterol (mmol/L) | 1.1 (0.3) | 1.2 (0.3) | 1.2 (0.4) | 1.1 (0.4) |
| Triglycerides (mmol/L) | 1.4 [1.0, 1.9] | 1.3 [1.0, 1.9] | 1.3 [0.9, 2.0] | 1.2 [0.9, 1.8] |
| Haemoglobin (g/L) | 143.3 (16.5) | 136.2 (19.5) | 137.6 (19.6) | 127.4 (21.8) |
| Glucose (mmol/L) | 6.7 [5.7, 8.4] | 6.5 [5.5, 8.2] | 6.3 [5.4, 8.0] | 7.1 [5.8, 9.4] |
| Electronic frailty index** (%) |  |  |  |  |
| No frailty | 2,903 (70.4%) | 1,032 (54.0%) | 174 (60.6%) | 151 (30.1%) |
| Mild frailty | 997 (24.2%) | 635 (33.2%) | 84 (29.3%) | 212 (42.3%) |
| Moderate frailty | 189 (4.6%) | 201 (10.5%) | 26 (9.1%) | 102 (20.4%) |
| Severe frailty | 32 (0.8%) | 44 (2.3%) | <5 | 36 (7.2%) |
| **Medications prior to index event** |  |  |  |  |
| Antithrombotic agent (%) | 674 (13.8%) | 427 (20.0%) | 42 (12.6%) | 206 (36.9%) |
| Lipid-lowering therapy (%) | 1,137 (23.2%) | 453 (21.2%) | 42 (12.6%) | 181 (32.4%) |
| Renin-angiotensin system blocker (%) | 1,278 (26.1%) | 415 (19.4%) | 38 (11.4%) | 164 (29.3%) |
| **Index event characteristics** |  |  |  |  |
| Discharge diagnosis (%) |  |  |  |  |
| STEMI | 1,734 (46.6%) | 448 (27.4%) | 78 (31.1%) | 117 (28.4%) |
| NSTEMI | 1,989 (53.4%) | 1,186 (72.6%) | 173 (68.9%) | 295 (71.6%) |
| Coronary artery bypass graft (%) | 140 (2.9%) | 80 (3.7%) | <5 | 5 (0.9%) |
| Percutaneous coronary intervention (%) | 3,725 (76.1%) | 1,098 (51.3%) | 155 (46.5%) | 172 (30.8%) |
| Continuous variables are presented as mean (standard deviation) or median (25^th^ to 75^th^ percentile), as appropriate. Categorical variables are presented as number (%). Missing values <5% if applicable, except for ethnicity (8.3%), current smoker (13.5%), body mass index (28.8%), systolic blood pressure (10.8%), diastolic blood pressure (10.8%), cholesterol (23.2%), low-density lipoprotein (36.2%), high-density lipoprotein (34.6%), triglycerides (33.9%), glucose (18.9%), electronic frailty index (13.9%), and discharge diagnosis (24.0%). Abbreviations: STEMI, ST segment elevation myocardial infarction; NSTEMI, non-ST segment elevation myocardial infarction; TIA, transient ischaemic attack.  * Socioeconomic deprivation is determined using the Scottish Index of Multiple Deprivation (SIMD). The SIMD is an area-based measure of relative deprivation. Patients were assigned a quintile based on their individual SIMD rank and were divided into three subgroups: Group 1 (quintile 1) representing the most deprived 20% of the population, Group 2 (quintile 2 to 4), and Group 3 (quintile 5) representing the least deprived 20% of the population. ** The electronic frailty index is a tool that uses routinely collected primary care data to assess and quantify an individual's level of frailty based on the accumulation of health deficits. A higher score on the electronic frailty index indicates a higher level of frailty. Patients were divided into 4 groups based on their electronic frailty index: Fit or no frailty (frailty index 0 to 0.12), Mild frailty (frailty index >0.12 to 0.24), Moderate frailty (frailty index >0.24 to 0.36), and Severe frailty (frailty index >0.36). | | | | |

**Table S4.** **Baseline characteristics of patients with myocardial infarction by guideline-medical therapy status at 18 months.**

|  | **Guideline-directed medical therapy at 18 months** | | | |
| --- | --- | --- | --- | --- |
| **Characteristic** | **Three n = 4691** | **One/two n = 2118** | **None n = 402** | **Died or censored n = 715** |
| Age (years) | 62.0 (11.6) | 67.0 (14.1) | 62.8 (15.8) | 74.8 (14.1) |
| Sex (% male) | 3,302 (70.4%) | 1,183 (55.9%) | 232 (57.7%) | 407 (56.9%) |
| Ethnicity (%) |  |  |  |  |
| White | 4,157 (96.6%) | 1,924 (97.0%) | 344 (95.8%) | 608 (97.9%) |
| Asian/Black/Mixed/Other | 148 (3.4%) | 59 (3.0%) | 15 (4.2%) | 13 (2.1%) |
| Socioeconomic deprivation* (%) |  |  |  |  |
| Group 1 (most deprived) | 786 (16.8%) | 371 (17.5%) | 80 (20.1%) | 117 (16.4%) |
| Group 2 | 2,753 (58.9%) | 1,218 (57.6%) | 227 (56.9%) | 422 (59.2%) |
| Group 3 (least deprived) | 1,138 (24.3%) | 525 (24.8%) | 92 (23.1%) | 174 (24.4%) |
| History of diabetes (%) | 671 (14.3%) | 337 (15.9%) | 49 (12.2%) | 181 (25.3%) |
| History of hypertension (%) | 1,826 (38.9%) | 874 (41.3%) | 137 (34.1%) | 437 (61.1%) |
| History of obesity (%) | 880 (18.8%) | 389 (18.4%) | 58 (14.4%) | 150 (21.0%) |
| History of stroke and/or TIA (%) | 237 (5.1%) | 190 (9.0%) | 23 (5.7%) | 141 (19.7%) |
| History of heart failure (%) | 95 (2.0%) | 94 (4.4%) | 13 (3.2%) | 109 (15.2%) |
| Current smoker (%) | 1,594 (39.8%) | 638 (34.1%) | 146 (43.8%) | 161 (24.8%) |
| Body mass index (kg/m2) | 28.7 [25.5, 32.3] | 27.1 [23.9, 30.9] | 26.5 [22.8, 30.9] | 25.9 [22.6, 30.1] |
| Systolic blood pressure (mmHg) | 137.3 (17.4) | 135.9 (18.1) | 137.7 (20.1) | 136.8 (20.9) |
| Diastolic blood pressure (mmHg) | 80.6 (11.2) | 78.1 (11.0) | 79.9 (12.8) | 76.5 (12.1) |
| Estimated glomerular filtration rate (% yes) |  |  |  |  |
| <30 ml/min/1.73m2 | 30 (0.6%) | 71 (3.4%) | 9 (2.3%) | 78 (11.1%) |
| 30 to 44 ml/min/1.73m2 | 110 (2.4%) | 136 (6.5%) | 15 (3.8%) | 97 (13.8%) |
| 45 to 59 ml/min/1.73m2 | 315 (6.8%) | 204 (9.8%) | 29 (7.4%) | 128 (18.2%) |
| ≥ 60 ml/min/1.73m2 | 4,185 (90.2%) | 1,671 (80.3%) | 338 (86.4%) | 401 (57.0%) |
| Cholesterol (mmol/L) | 5.2 (1.3) | 5.1 (1.3) | 5.2 (1.2) | 4.6 (1.2) |
| Low-density lipoprotein cholesterol (mmol/L) | 3.2 (1.1) | 3.2 (1.1) | 3.2 (1.0) | 2.9 (1.1) |
| High-density lipoprotein cholesterol (mmol/L) | 1.1 (0.3) | 1.2 (0.3) | 1.2 (0.4) | 1.1 (0.4) |
| Triglycerides (mmol/L) | 1.4 [1.0, 1.9] | 1.3 [0.9, 1.9] | 1.4 [1.0, 2.1] | 1.2 [0.9, 1.8] |
| Haemoglobin (g/L) | 143.4 (16.4) | 136.8 (19.1) | 139.0 (19.9) | 127.9 (21.9) |
| Glucose (mmol/L) | 6.7 [5.7, 8.3] | 6.5 [5.5, 8.3] | 6.4 [5.4, 8.1] | 7.1 [5.8, 9.4] |
| Electronic frailty index** (%) |  |  |  |  |
| No frailty | 2,814 (71.4%) | 1,034 (55.0%) | 216 (61.9%) | 196 (30.2%) |
| Mild frailty | 936 (23.7%) | 621 (33.0%) | 103 (29.5%) | 268 (41.4%) |
| Moderate frailty | 165 (4.2%) | 188 (10.0%) | 24 (6.9%) | 141 (21.8%) |
| Severe frailty | 28 (0.7%) | 38 (2.0%) | 6 (1.7%) | 43 (6.6%) |
| **Medications prior to index event** |  |  |  |  |
| Antithrombotic agent (%) | 633 (13.5%) | 406 (19.2%) | 43 (10.7%) | 267 (37.3%) |
| Lipid-lowering therapy (%) | 1,076 (22.9%) | 453 (21.4%) | 53 (13.2%) | 231 (32.3%) |
| Renin-angiotensin system blocker (%) | 1,188 (25.3%) | 439 (20.7%) | 63 (15.7%) | 205 (28.7%) |
| **Index event characteristics** |  |  |  |  |
| Discharge diagnosis (%) |  |  |  |  |
| STEMI | 1,657 (46.5%) | 455 (28.2%) | 102 (33.4%) | 163 (30.5%) |
| NSTEMI | 1,910 (53.5%) | 1,158 (71.8%) | 203 (66.6%) | 372 (69.5%) |
| Coronary artery bypass graft (%) | 131 (2.8%) | 81 (3.8%) | 7 (1.7%) | 8 (1.1%) |
| Percutaneous coronary intervention (%) | 3,603 (76.8%) | 1,116 (52.7%) | 196 (48.8%) | 235 (32.9%) |
| Continuous variables are presented as mean (standard deviation) or median (25^th^ to 75^th^ percentile), as appropriate. Categorical variables are presented as number (%). Missing values <5% if applicable, except for ethnicity (8.3%), current smoker (13.5%), body mass index (28.8%), systolic blood pressure (10.8%), diastolic blood pressure (10.8%), cholesterol (23.2%), low-density lipoprotein (36.2%), high-density lipoprotein (34.6%), triglycerides (33.9%), glucose (18.9%), electronic frailty index (13.9%), and discharge diagnosis (24.0%). Abbreviations: STEMI, ST segment elevation myocardial infarction; NSTEMI, non-ST segment elevation myocardial infarction; TIA, transient ischaemic attack.  * Socioeconomic deprivation is determined using the Scottish Index of Multiple Deprivation (SIMD). The SIMD is an area-based measure of relative deprivation. Patients were assigned a quintile based on their individual SIMD rank and were divided into three subgroups: Group 1 (quintile 1) representing the most deprived 20% of the population, Group 2 (quintile 2 to 4), and Group 3 (quintile 5) representing the least deprived 20% of the population. ** The electronic frailty index is a tool that uses routinely collected primary care data to assess and quantify an individual's level of frailty based on the accumulation of health deficits. A higher score on the electronic frailty index indicates a higher level of frailty. Patients were divided into 4 groups based on their electronic frailty index: Fit or no frailty (frailty index 0 to 0.12), Mild frailty (frailty index >0.12 to 0.24), Moderate frailty (frailty index >0.24 to 0.36), and Severe frailty (frailty index >0.36). | | | | |

**Table S5. Baseline characteristics of patients with myocardial infarction by sex.**

| **Characteristic** | **Female n = 2802 (35%)** | **Male n = 5124 (65%)** |
| --- | --- | --- |
| Age (years) | 68.9 (13.5) | 62.1 (12.6) |
| Ethnicity (%) |  |  |
| White | 2,522 (97.7%) | 4,511 (96.3%) |
| Asian/Black/Mixed/Other | 60 (2.3%) | 175 (3.7%) |
| Socioeconomic deprivation* (%) |  |  |
| Group 1 (most deprived) | 531 (19.0%) | 823 (16.1%) |
| Group 2 | 1,601 (57.3%) | 3,019 (59.1%) |
| Group 3 (least deprived) | 663 (23.7%) | 1,266 (24.8%) |
| History of diabetes (%) | 458 (16.3%) | 780 (15.2%) |
| History of hypertension (%) | 1,378 (49.2%) | 1,896 (37.0%) |
| History of obesity (%) | 615 (21.9%) | 862 (16.8%) |
| History of stroke and/or TIA (%) | 266 (9.5%) | 325 (6.3%) |
| History of heart failure (%) | 148 (5.3%) | 163 (3.2%) |
| Current smoker (%) | 892 (35.3%) | 1,647 (38.0%) |
| Body mass index (kg/m2) | 27.2 [23.5, 31.5] | 28.3 [25.2, 31.8] |
| Systolic blood pressure (mmHg) | 137.3 (19.0) | 136.7 (17.5) |
| Diastolic blood pressure (mmHg) | 77.8 (11.2) | 80.4 (11.4) |
| Estimated glomerular filtration rate (% yes) |  |  |
| <30 ml/min/1.73m2 | 90 (3.2%) | 98 (1.9%) |
| 30 to 44 ml/min/1.73m2 | 197 (7.1%) | 161 (3.2%) |
| 45 to 59 ml/min/1.73m2 | 337 (12.2%) | 339 (6.7%) |
| ≥ 60 ml/min/1.73m2 | 2,147 (77.5%) | 4,448 (88.1%) |
| Cholesterol (mmol/L) | 5.3 (1.4) | 5.0 (1.3) |
| Low-density lipoprotein cholesterol (mmol/L) | 3.3 (1.2) | 3.2 (1.1) |
| High-density lipoprotein cholesterol (mmol/L) | 1.3 (0.4) | 1.1 (0.3) |
| Triglycerides (mmol/L) | 1.3 [1.0, 1.8] | 1.4 [1.0, 1.9] |
| Haemoglobin (g/L) | 130.6 (16.5) | 145.2 (17.5) |
| Glucose (mmol/L) | 6.7 [5.6, 8.4] | 6.6 [5.6, 8.3] |
| Electronic frailty index** (%) |  |  |
| No frailty | 1,174 (46.8%) | 3,086 (71.6%) |
| Mild frailty | 940 (37.5%) | 988 (22.9%) |
| Moderate frailty | 315 (12.6%) | 203 (4.7%) |
| Severe frailty | 80 (3.2%) | 35 (0.8%) |
| **Medications prior to index event** |  |  |
| Antithrombotic agent (%) | 574 (20.5%) | 775 (15.1%) |
| Lipid-lowering therapy (%) | 695 (24.8%) | 1,118 (21.8%) |
| Renin-angiotensin system blocker (%) | 745 (26.6%) | 1,150 (22.4%) |
|  |  |  |
| **Index event characteristics** |  |  |
| Discharge diagnosis (%) |  |  |
| STEMI | 725 (34.3%) | 1,652 (42.3%) |
| NSTEMI | 1,388 (65.7%) | 2,255 (57.7%) |
| Coronary artery bypass graft (%) | 60 (2.1%) | 167 (3.3%) |
| Percutaneous coronary intervention (%) | 1,468 (52.4%) | 3,682 (71.9%) |
| Continuous variables are presented as mean (standard deviation) or median (25^th^ to 75^th^ percentile), as appropriate. Categorical variables are presented as number (%). Abbreviations: STEMI, ST segment elevation myocardial infarction; NSTEMI, non-ST segment elevation myocardial infarction; TIA, transient ischaemic attack.  * Socioeconomic l deprivation is determined using the Scottish Index of Multiple Deprivation (SIMD). The SIMD is an area-based measure of relative deprivation. Patients were assigned a quintile based on their individual SIMD rank and were divided into three subgroups: Group 1 (quintile 1) representing the most deprived 20% of the population, Group 2 (quintile 2 to 4), and Group 3 (quintile 5) representing the least deprived 20% of the population. ** The electronic frailty index is a tool that uses routinely collected primary care data to assess and quantify an individual's level of frailty based on the accumulation of health deficits. A higher score on the electronic frailty index indicates a higher level of frailty. Patients were divided into 4 groups based on their electronic frailty index: Fit or no frailty (frailty index 0 to 0.12), Mild frailty (frailty index >0.12 to 0.24), Moderate frailty (frailty index >0.24 to 0.36), and Severe frailty (frailty index >0.36). | | |

**Table S6. Baseline characteristics of patients with myocardial infarction by age into <50 years, 50 to 70 years, and >70 years groups.**

| **Characteristic** | **<50 years n = 1101 (14%)** | **50 to 70 years n = 4122 (52%)** | **>70 years n = 2703 (34%)** |
| --- | --- | --- | --- |
| Age (years) | 44.2 (4.5) | 60.2 (5.8) | 79.4 (6.3) |
| Sex (% male) | 851 (77.3%) | 2,911 (70.6%) | 1,362 (50.4%) |
| Ethnicity (%) |  |  |  |
| White | 970 (93.1%) | 3,645 (96.6%) | 2,418 (98.6%) |
| Asian/Black/Mixed/Other | 72 (6.9%) | 129 (3.4%) | 34 (1.4%) |
| Socioeconomic deprivation* (%) |  |  |  |
| Group 1 (most deprived) | 269 (24.5%) | 745 (18.1%) | 340 (12.6%) |
| Group 2 | 674 (61.4%) | 2,408 (58.6%) | 1,538 (57.0%) |
| Group 3 (least deprived) | 155 (14.1%) | 953 (23.2%) | 821 (30.4%) |
| History of diabetes (%) | 129 (11.7%) | 597 (14.5%) | 512 (18.9%) |
| History of hypertension (%) | 205 (18.6%) | 1,404 (34.1%) | 1,665 (61.6%) |
| History of obesity (%) | 187 (17.0%) | 800 (19.4%) | 490 (18.1%) |
| History of stroke and/or TIA (%) | 18 (1.6%) | 197 (4.8%) | 376 (13.9%) |
| History of heart failure (%) | 7 (0.6%) | 76 (1.8%) | 228 (8.4%) |
| Current smoker (%) | 611 (66.3%) | 1,536 (43.5%) | 392 (16.3%) |
| Body mass index (kg/m2) | 29.3 [25.3, 33.5] | 28.7 [25.6, 32.4] | 26.4 [23.3, 30.0] |
| Systolic blood pressure (mmHg) | 134.9 (17.6) | 136.4 (17.7) | 138.3 (18.7) |
| Diastolic blood pressure (mmHg) | 84.2 (11.7) | 81.1 (10.9) | 75.6 (10.8) |
| Estimated glomerular filtration rate (% yes) |  |  |  |
| <30 ml/min/1.73m2 | 6 (0.6%) | 44 (1.1%) | 138 (5.1%) |
| 30 to 44 ml/min/1.73m2 | 6 (0.6%) | 58 (1.4%) | 294 (11.0%) |
| 45 to 59 ml/min/1.73m2 | 17 (1.6%) | 195 (4.8%) | 464 (17.3%) |
| ≥ 60 ml/min/1.73m2 | 1,053 (97.3%) | 3,756 (92.7%) | 1,786 (66.6%) |
| Cholesterol (mmol/L) | 5.4 (1.3) | 5.2 (1.3) | 4.7 (1.2) |
| Low-density lipoprotein cholesterol (mmol/L) | 3.5 (1.1) | 3.3 (1.1) | 2.9 (1.0) |
| High-density lipoprotein cholesterol (mmol/L) | 1.0 (0.3) | 1.1 (0.3) | 1.2 (0.4) |
| Triglycerides (mmol/L) | 1.6 [1.1, 2.5] | 1.4 [1.0, 2.0] | 1.1 [0.9, 1.5] |
| Haemoglobin (g/L) | 147.5 (16.0) | 143.9 (16.7) | 131.2 (18.8) |
| Glucose (mmol/L) | 6.5 [5.5, 8.2] | 6.6 [5.6, 8.3] | 6.9 [5.7, 8.7] |
| Electronic frailty index** (% yes) |  |  |  |
| No frailty | 785 (87.7%) | 2,622 (75.7%) | 853 (34.6%) |
| Mild frailty | 105 (11.7%) | 716 (20.7%) | 1,107 (44.9%) |
| Moderate frailty | 5 (0.6%) | 114 (3.3%) | 399 (16.2%) |
| Severe frailty | <5 | 10 (0.3%) | 105 (4.3%) |
| **Medications prior to index event** |  |  |  |
| Antithrombotic agent (%) | 37 (3.4%) | 488 (11.8%) | 824 (30.5%) |
| Lipid-lowering therapy (%) | 105 (9.5%) | 868 (21.1%) | 840 (31.1%) |
| Renin-angiotensin system blocker (%) | 128 (11.6%) | 879 (21.3%) | 888 (32.9%) |
| **Index event characteristics** |  |  |  |
| Discharge diagnosis (%) |  |  |  |
| STEMI | 379 (45.7%) | 1,335 (41.8%) | 663 (33.2%) |
| NSTEMI | 450 (54.3%) | 1,861 (58.2%) | 1,332 (66.8%) |
| Coronary artery bypass graft (%) | 10 (0.9%) | 135 (3.3%) | 82 (3.0%) |
| Percutaneous coronary intervention (%) | 845 (76.7%) | 3,057 (74.2%) | 1,248 (46.2%) |
| Continuous variables are presented as mean (standard deviation) or median (25^th^ to 75^th^ percentile), as appropriate. Categorical variables are presented as number (%). Abbreviations: STEMI, ST segment elevation myocardial infarction; NSTEMI, non-ST segment elevation myocardial infarction; TIA, transient ischaemic attack.  * Socioeconomic deprivation is determined using the Scottish Index of Multiple Deprivation (SIMD). The SIMD is an area-based measure of relative deprivation. Patients were assigned a quintile based on their individual SIMD rank and were divided into three subgroups: Group 1 (quintile 1) representing the most deprived 20% of the population, Group 2 (quintile 2 to 4), and Group 3 (quintile 5) representing the least deprived 20% of the population. ** The electronic frailty index is a tool that uses routinely collected primary care data to assess and quantify an individual's level of frailty based on the accumulation of health deficits. A higher score on the electronic frailty index indicates a higher level of frailty. Patients were divided into 4 groups based on their electronic frailty index: Fit or no frailty (frailty index 0 to 0.12), Mild frailty (frailty index >0.12 to 0.24), Moderate frailty (frailty index >0.24 to 0.36), and Severe frailty (frailty index >0.36). | | | |

**Table S7. Baseline characteristics of patients with myocardial infarction by ethnicity into White and Asian/Black/Mixed/Other groups.**

| **Characteristic** | **White n = 7033 (97%)** | **Asian/Black/Mixed/Other n = 235 (3.2%)** |
| --- | --- | --- |
| Age (years) | 64.6 (13.3) | 56.8 (12.7) |
| Sex (% male) | 4,511 (64.1%) | 175 (74.5%) |
| Socioeconomic deprivation* (%) |  |  |
| Group 1 (most deprived) | 1,244 (17.7%) | 44 (18.7%) |
| Group 2 | 4,149 (59.2%) | 109 (46.4%) |
| Group 3 (least deprived) | 1,617 (23.1%) | 82 (34.9%) |
| History of diabetes (%) | 1,084 (15.4%) | 73 (31.1%) |
| History of hypertension (%) | 2,910 (41.4%) | 94 (40.0%) |
| History of obesity (%) | 1,353 (19.2%) | 43 (18.3%) |
| History of stroke and/or TIA (%) | 543 (7.7%) | 11 (4.7%) |
| History of heart failure (%) | 275 (3.9%) | 11 (4.7%) |
| Current smoker (%) | 2,289 (37.4%) | 67 (33.7%) |
| Body mass index (kg/m2) | 28.0 [24.6, 31.7] | 27.6 [24.6, 30.8] |
| Systolic blood pressure (mmHg) | 136.9 (18.0) | 136.2 (19.8) |
| Diastolic blood pressure (mmHg) | 79.4 (11.4) | 81.1 (12.8) |
| Estimated glomerular filtration rate (% yes) |  |  |
| <30 ml/min/1.73m2 | 155 (2.2%) | 11 (4.7%) |
| 30 to 44 ml/min/1.73m2 | 333 (4.8%) | <5 |
| 45 to 59 ml/min/1.73m2 | 594 (8.6%) | 15 (6.5%) |
| ≥ 60 ml/min/1.73m2 | 5,859 (84.4%) | 202 (87.1%) |
| Cholesterol (mmol/L) | 5.1 (1.3) | 5.1 (1.3) |
| Low-density lipoprotein cholesterol (mmol/L) | 3.2 (1.1) | 3.1 (1.1) |
| High-density lipoprotein cholesterol (mmol/L) | 1.1 (0.3) | 1.1 (0.3) |
| Triglycerides (mmol/L) | 1.4 [1.0, 1.9] | 1.5 [1.1, 2.3] |
| Haemoglobin (g/L) | 140.2 (18.4) | 136.9 (19.3) |
| Glucose (mmol/L) | 6.6 [5.6, 8.4] | 7.4 [5.9, 10.2] |
| Electronic frailty index** (%) |  |  |
| No frailty | 3,730 (61.5%) | 146 (74.9%) |
| Mild frailty | 1,732 (28.6%) | 39 (20.0%) |
| Moderate frailty | 490 (8.1%) | 8 (4.1%) |
| Severe frailty | 110 (1.8%) | <5 |
| **Medications prior to index event** |  |  |
| Antithrombotic agent (%) | 1,212 (17.2%) | 37 (15.7%) |
| Lipid-lowering therapy (%) | 1,624 (23.1%) | 66 (28.1%) |
| Renin-angiotensin system blocker (%) | 1,690 (24.0%) | 49 (20.9%) |
|  |  |  |
| **Index event characteristics** |  |  |
| Discharge diagnosis (%) |  |  |
| STEMI | 2,106 (39.2%) | 65 (36.7%) |
| NSTEMI | 3,271 (60.8%) | 112 (63.3%) |
| Coronary artery bypass graft (%) | 209 (3.0%) | <5 |
| Percutaneous coronary intervention (%) | 4,557 (64.8%) | 156 (66.4%) |
| Continuous variables are presented as mean (standard deviation) or median (25^th^ to 75^th^ percentile), as appropriate. Categorical variables are presented as number (%). Abbreviations: STEMI, ST segment elevation myocardial infarction; NSTEMI, non-ST segment elevation myocardial infarction; TIA, transient ischaemic attack.  * Socioeconomic deprivation is determined using the Scottish Index of Multiple Deprivation (SIMD). The SIMD is an area-based measure of relative deprivation. Patients were assigned a quintile based on their individual SIMD rank and were divided into three subgroups: Group 1 (quintile 1) representing the most deprived 20% of the population, Group 2 (quintile 2 to 4), and Group 3 (quintile 5) representing the least deprived 20% of the population. ** The electronic frailty index is a tool that uses routinely collected primary care data to assess and quantify an individual's level of frailty based on the accumulation of health deficits. A higher score on the electronic frailty index indicates a higher level of frailty. Patients were divided into 4 groups based on their electronic frailty index: Fit or no frailty (frailty index 0 to 0.12), Mild frailty (frailty index >0.12 to 0.24), Moderate frailty (frailty index >0.24 to 0.36), and Severe frailty (frailty index >0.36). | | |

**Table S8. Baseline characteristics of patients with myocardial infarction by socioeconomic deprivation status into group 1 (most deprived), group 2 and group 3 (least deprived).**

| **Characteristic** | **Group 1 (most deprived) n = 1354 (17%)** | **Group 2 n = 4620 (58%)** | **Group 3 (least deprived) n = 1929 (24%)** |
| --- | --- | --- | --- |
| Age (years) | 61.2 (12.9) | 64.2 (13.3) | 67.7 (12.9) |
| Sex (% male) | 823 (60.8%) | 3,019 (65.3%) | 1,266 (65.6%) |
| Ethnicity (%) |  |  |  |
| White | 1,244 (96.6%) | 4,149 (97.4%) | 1,617 (95.2%) |
| Asian/Black/Mixed/Other | 44 (3.4%) | 109 (2.6%) | 82 (4.8%) |
| History of diabetes (%) | 270 (19.9%) | 739 (16.0%) | 227 (11.8%) |
| History of hypertension (%) | 534 (39.4%) | 1,905 (41.2%) | 830 (43.0%) |
| History of obesity (%) | 341 (25.2%) | 880 (19.0%) | 254 (13.2%) |
| History of stroke and/or TIA (%) | 98 (7.2%) | 342 (7.4%) | 148 (7.7%) |
| History of heart failure (%) | 56 (4.1%) | 182 (3.9%) | 72 (3.7%) |
| Current smoker (%) | 640 (52.6%) | 1,568 (38.5%) | 320 (20.7%) |
| Body mass index (kg/m2) | 28.4 [24.8, 32.5] | 28.1 [24.6, 31.9] | 27.4 [24.5, 30.4] |
| Systolic blood pressure (mmHg) | 136.4 (18.8) | 137.1 (18.3) | 136.8 (17.2) |
| Diastolic blood pressure (mmHg) | 79.9 (12.1) | 79.4 (11.4) | 79.2 (11.0) |
| Estimated glomerular filtration rate (% yes) |  |  |  |
| <30 ml/min/1.73m2 | 31 (2.3%) | 116 (2.5%) | 41 (2.2%) |
| 30 to 44 ml/min/1.73m2 | 59 (4.4%) | 218 (4.8%) | 79 (4.2%) |
| 45 to 59 ml/min/1.73m2 | 106 (7.9%) | 405 (8.9%) | 164 (8.6%) |
| ≥ 60 ml/min/1.73m2 | 1,145 (85.4%) | 3,816 (83.8%) | 1,615 (85.0%) |
| Cholesterol (mmol/L) | 5.1 (1.4) | 5.1 (1.3) | 5.1 (1.2) |
| Low-density lipoprotein cholesterol (mmol/L) | 3.2 (1.1) | 3.2 (1.1) | 3.2 (1.1) |
| High-density lipoprotein cholesterol (mmol/L) | 1.1 (0.3) | 1.1 (0.3) | 1.2 (0.4) |
| Triglycerides (mmol/L) | 1.5 [1.0, 2.1] | 1.4 [1.0, 1.9] | 1.3 [0.9, 1.8] |
| Haemoglobin (g/L) | 140.6 (18.4) | 140.0 (18.6) | 139.6 (18.5) |
| Glucose (mmol/L) | 6.9 [5.7, 8.9] | 6.6 [5.6, 8.4] | 6.5 [5.5, 8.2] |
| Electronic frailty index** (%) |  |  |  |
| No frailty | 691 (58.6%) | 2,490 (62.3%) | 1,062 (65.6%) |
| Mild frailty | 349 (29.6%) | 1,126 (28.2%) | 447 (27.6%) |
| Moderate frailty | 111 (9.4%) | 308 (7.7%) | 99 (6.1%) |
| Severe frailty | 29 (2.5%) | 75 (1.9%) | 11 (0.7%) |
| **Medications prior to index event** |  |  |  |
| Antithrombotic agent (%) | 219 (16.2%) | 788 (17.1%) | 338 (17.5%) |
| Lipid-lowering therapy (%) | 337 (24.9%) | 1,070 (23.2%) | 402 (20.8%) |
| Renin-angiotensin system blocker (%) | 327 (24.2%) | 1,124 (24.3%) | 441 (22.9%) |
| **Index event characteristics** |  |  |  |
| Discharge diagnosis (%) |  |  |  |
| STEMI | 410 (40.1%) | 1,418 (40.3%) | 539 (36.7%) |
| NSTEMI | 613 (59.9%) | 2,099 (59.7%) | 928 (63.3%) |
| Coronary artery bypass graft (%) | 45 (3.3%) | 136 (2.9%) | 46 (2.4%) |
| Percutaneous coronary intervention (%) | 887 (65.5%) | 3,011 (65.2%) | 1,235 (64.0%) |
| Continuous variables are presented as mean (standard deviation) or median (25^th^ to 75^th^ percentile), as appropriate. Categorical variables are presented as number (%). Abbreviations: STEMI, ST segment elevation myocardial infarction; NSTEMI, non-ST segment elevation myocardial infarction; TIA, transient ischaemic attack.  * Socioeconomic deprivation is determined using the Scottish Index of Multiple Deprivation (SIMD). The SIMD is an area-based measure of relative deprivation. Patients were assigned a quintile based on their individual SIMD rank and were divided into three subgroups: Group 1 (quintile 1) representing the most deprived 20% of the population, Group 2 (quintile 2 to 4), and Group 3 (quintile 5) representing the least deprived 20% of the population. ** The electronic frailty index is a tool that uses routinely collected primary care data to assess and quantify an individual's level of frailty based on the accumulation of health deficits. A higher score on the electronic frailty index indicates a higher level of frailty. Patients were divided into 4 groups based on their electronic frailty index: Fit or no frailty (frailty index 0 to 0.12), Mild frailty (frailty index >0.12 to 0.24), Moderate frailty (frailty index >0.24 to 0.36), and Severe frailty (frailty index >0.36). | | | |

**Table S9. Crude event rates by sex, ethnicity, and socioeconomic deprivation groups.** Median follow-up was 58.4 [25^th^ percentile, 75^th^ percentile, 28.7, 100.1] months.

| **Subgroup** | **Cumulative incidence at median follow-up since discharge** |
| --- | --- |
| Men | 13.5% |
| Women | 14.8% |
| <50 years | 11.1% |
| 50 to 70 years | 11.1% |
| >70 years | 19.4% |
| White | 14.2% |
| Asian/Black/Mixed/Other | 12.0% |
| Group 1 (most deprived) | 15.3% |
| Group 2 | 13.6% |
| Group 3 (least deprived) | 13.8% |
